# Stability and Reversible Oxidation of Sub‐Nanometric Cu_5_ Metal Clusters: Integrated Experimental Study and Theoretical Modeling**

**DOI:** 10.1002/chem.202301517

**Published:** 2023-07-07

**Authors:** David Buceta, Shahana Huseyinova, Miguel Cuerva, Héctor Lozano, Lisandro J. Giovanetti, José M. Ramallo‐López, Patricia López‐Caballero, Alexandre Zanchet, Alexander O. Mitrushchenkov, Andreas W. Hauser, Giampaolo Barone, Cristián Huck‐Iriart, Carlos Escudero, Juan Carlos Hernández‐Garrido, José Juan Calvino, Miguel López‐Haro, María Pilar de Lara‐Castells, Félix G. Requejo, M. Arturo López‐Quintela

**Affiliations:** ^1^ Department of Physical Chemistry, Nanomag Laboratory Universidad de Santiago de Compostela 15782 Santiago de Compostela Spain; ^2^ Instituto de Investigaciones Fisicoquímicas Teóricas y Aplicadas (INIFTA) Dto. de Química, Facultad de Ciencias Exactas, UNLP and CONICET Diag. 113 y 64. 1900 La Plata Argentina; ^3^ Instituto de Física Fundamental (AbinitSim Unit) CSIC Serrano 123 28006 Madrid Spain; ^4^ MSME Univ Gustave Eiffel, UPEC, CNRS 77454 Marne-la-Vallée France; ^5^ Institute of Experimental Physics Graz University of Technology Petersgasse 16 8010 Graz Austria; ^6^ Department of Biological, Chemical and Pharmaceutical Sciences and Technologies University of Palermo 90128 Palermo Italy; ^7^ Laboratorio de Cristalografía Aplicada Escuela de Ciencia y Tecnología Universidad Nacional de San Martín (UNSAM) Campus Miguelete, 25 de Mayo y Francia 1650 San Martín, Provincia Buenos Aires Argentina; ^8^ ALBA Synchrotron Light Source Carrer de la Llum 2–26 08290 Cerdanyola del Vallès Barcelona Spain; ^9^ Department of Material Science and Metallurgic Engineering and Inorganic Chemistry Faculty of Science University of Cádiz 11510 Puerto Real Cádiz Spain

**Keywords:** density functional calculations, nanotechnology, oxidation, photoelectron spectroscopy, X-ray absorption spectroscopy

## Abstract

Sub‐nanometer metal clusters have special physical and chemical properties, significantly different from those of nanoparticles. However, there is a major concern about their thermal stability and susceptibility to oxidation. In situ X‐ray Absorption spectroscopy and Near Ambient Pressure X‐ray Photoelectron spectroscopy results reveal that supported Cu_5_ clusters are resistant to irreversible oxidation at least up to 773 K, even in the presence of 0.15 mbar of oxygen. These experimental findings can be formally described by a theoretical model which combines dispersion‐corrected DFT and first principles thermochemistry revealing that most of the adsorbed O_2_ molecules are transformed into superoxo and peroxo species by an interplay of collective charge transfer within the network of Cu atoms and large amplitude “breathing” motions. A chemical phase diagram for Cu oxidation states of the Cu_5_‐oxygen system is presented, clearly different from the already known bulk and nano‐structured chemistry of Cu.

## Graphical abstract

A combined XANES and NAP XPS study shows that Cu_5_ clusters are resistant to irreversible oxidation up to 773 K in the presence of oxygen. A theoretical model reveals that adsorbed O_2_ molecules are transformed into superoxo and peroxo species by an interplay of collective charge transfer and large amplitude “breathing” motions. A chemical phase diagram for Cu oxidation state of the Cu_5_‐O_2_ system is presented.

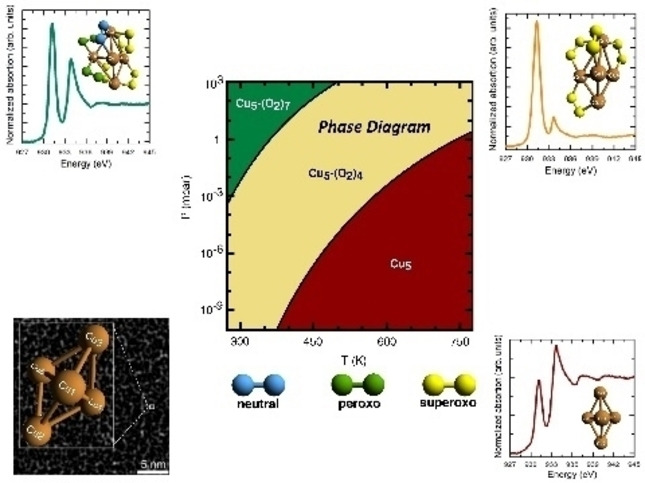

## Introduction

Geometry and electronic structure of transition‐metal nanoparticles change drastically when the size is reduced below 1–1.5 nm (i. e., below 100–150 atoms) due to quantum confinement effects.[^1^, ^2^] In this size regime the metallic band structure breaks into a series of discrete electronic levels. This change gives rise to novel properties which differ from those of larger nanomaterials or bulk.^[3]^ Due to the discretization of energy levels, sub‐nanometer‐sized metal clusters act as atomic‐scale semiconductors and collective phenomena such as the localized plasmon resonance absorption (LSPR), exhibited by larger metallic nanoparticles,^[4]^ cannot take place. In particular, when the cluster is composed of a very small number of atoms, a molecular network of *d*‐type orbitals interconnects the metal atoms, with the inter‐atomic distances having the length of a chemical bond (1‐2 Å). The “floppy” character of the resulting structures leads to the property of structural fluxionality,[^5^, ^6^, ^7^, ^8^, ^9^] a feature with the potential to enhance catalytic activity.^[5]^ Recently, sub‐nanometer copper‐based materials have attracted much interest in the field of catalysis.^[10]^ In particular, copper clusters are catalytically active in the oxidation of CO,[^11^, ^12^] the reduction of CO,[^2^, ^5^] the selective hydrogenation of olefin and carbonyl groups,[^13^, ^14^] or in C−X (being X=C, N, S, P) bond forming reactions.^[15]^ When these clusters are supported on titanium dioxide,^[16]^ photon energy is temporarily stored in the form of charge pairs in the direct vicinity of the surface which is a prerequisite for follow‐up chemistry.[^5^, ^17^] Moreover, it has been observed that sub‐nanometer structures are able to catalyze reactions at lower temperatures and pressures compared to bulk and conventional nanosized materials.^[18]^ Further experiments, performed by in situ Cu K‐edge XANES, explored the reactivity of sub‐nanometer Cu_n_O_x_ clusters.[^19^, ^20^, ^21^] These studies refer to chemical and thermodynamic properties of supported clusters on surfaces with strong support interaction as alumina or zirconia. Mammen et al.^[19]^ explore the oxidation of supported clusters on a hydroxylated amorphous alumina substrate in an O_2_‐rich environment at different temperatures, showing that the smaller the cluster, the greater is the tendency toward oxidation, but they do not report the reversibility of the process.

In spite of the known susceptibility to oxidation observed in Cu nanoparticles, ex‐situ measurements^[10]^ and theoretical studies considering the adsorption of one oxygen molecule[^22^, ^23^] have indicated the possibility of reversible oxidation of sub‐nanometer Cu clusters at temperatures below 423 K.^[23]^ However, up to date, the quenching of oxidation of these clusters under oxidative conditions has remained an open question due to the lack of in‐situ experiments under such experimental conditions. To achieve direct experimental evidence on the oxidation quenching of Cu clusters and to infer the associated mechanism, we conducted a quantitative experimental investigation of well‐defined bare Cu clusters of five atoms (Cu_5_) that are synthesized by a modified version of a previously reported electrochemical method.^[24]^ The latter allows their production with high concentrations, a requirement for accurate spectroscopic characterizations. Using a combination of experimental techniques (in‐situ X‐ray Absorption Near Edge Spectroscopy (XANES) and Near‐Ambient Pressure X‐ray Photoelectron Spectroscopy (NAP‐XPS)) on Cu_5_ clusters supported on Highly Oriented Pyrolytic Graphite (HOPG) we surprisingly found that, depending on the experimental conditions, clusters are not irreversibly oxidized even at temperatures as high as 773 K. Using dispersion‐corrected DFT and first principles thermochemistry, and applying high level ab initio theory we could reveal the mechanism associated with this unexpected behavior, which is based on the reversible interaction between Cu_5_ clusters and O_2_. We have further identified the activated oxygen species which are formed depending on the thermodynamic conditions of temperature and oxygen pressure. Such reversible O_2_ adsorption is the result of concerted and wide amplitude rearrangements of the atomic nuclei and coordinated charge transfer processes within a network of Cu 3*d* orbitals. Our results show not only the large stability of Cu_5_ clusters, but also the ability to activate O_2_ what is very important to understand and guide the applications of such clusters in the catalysis of oxidation reactions.

## Results and Discussion

### Synthesis and STEM characterization

**Figure 1:**
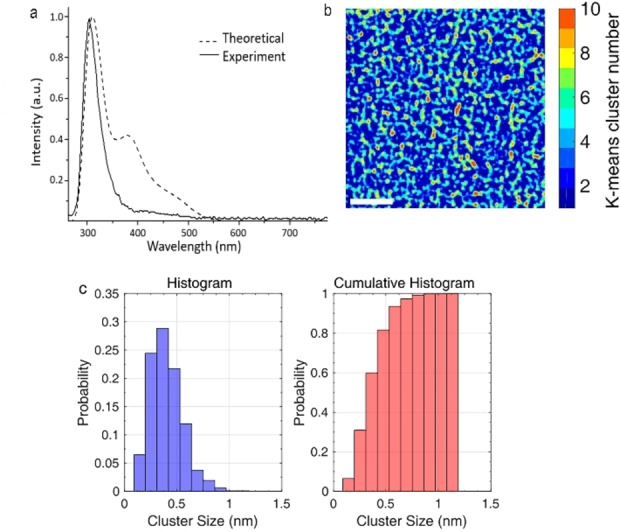
Characterization of the synthesized Cu clusters. a, Experimental emission spectrum (excitation at λ=224 nm) of the synthesized Cu clusters (continuous line) and the theoretical emission predicted for 3D Cu_5_ clusters## (dotted line). b, *k*‐means clustering result from experimental AC‐STEM‐HAADF image of Cu_5_ after denoising and background subtraction. c, Clusters size distribution histogram obtained after clustering and segmentation. The error bar derives from estimating the average cluster size after eroding (−) and dilating (+) by 1 pixel each object in the whole set of binarized images.

The synthesis of Cu_5_ was carried out by a modified version of a previously reported electrochemical method,^[24]^ allowing the production of monodisperse clusters with the high concentrations required for the study, (in the range ≈40 mg/L) (for details see Supporting Information). A water‐based dispersion of clusters displays only one main emission peak at 305 nm, which agrees with the previously reported one for Cu_5_ clusters synthesized by a similar electrochemical procedure^[24]^ and with the theoretically predicted, as it can be seen in Figure 1a. The emission peak can be used to obtain the HOMO‐LUMO gap of the synthesized clusters (≈4.07 eV). This gap is similar to the theoretical predicted for Cu_5_ clusters in a trapezoidal shape (4.58 eV).^[25]^ Moreover, the estimated cluster size agrees also with the Jellium model prediction, which represents a good approximation for clusters without strong binding ligands (see for example Refs. [24] [26]), from the equation *N*_atoms_=(E_*F*_/E_*g*_)^3^≈5 (with E_*F*_ denoting the Cu Fermi level at 7.0 eV, and E_*g*_ the HOMO‐LUMO gap, approximated by the emission peak). All of this confirms the presence of a highly monodispersed sample of Cu clusters consisting of 5 atoms.

Figures 1b‐c show the results of an Aberration‐Corrected Scanning Transmission Electron Microscopy study, working in the High Angle Annular Dark Field imaging mode (AC‐STEM‐HAADF), of the clusters at very low concentration (≈10 ng/mL, corresponding to less than 1 monolayer). The visualization of the Cu_5_ has been improved by feeding raw image data to an advanced image processing pipeline including denoising and background subtraction (see Supporting Information Figure 1). To determine, in a fully automated, user‐independent, and statistically meaningful way, the size of the clusters observed in the experimental images, a segmentation based on *k*‐means clustering techniques was performed (see Figure 1b, Supporting Information Figures 2a–c). To validate this analysis, HAADF‐STEM images were calculated for models of Cu_3_ and Cu_5_ clusters, in the last case considering both planar (2D) and trigonal bipyramidal (3D) structures (see Supporting Information Figure 2d). Then, the histogram and cumulative histogram from five different experimental HAADF‐STEM binarized images were calculated (Figure 1c). The analysis indicates that about 85 % of the Cu clusters in this sample are below 0.5 nm in size. According to the luminescence results, the remaining part of the distribution, with size in the range 0.5–0.9 nm, should correspond to the superposition of neighboring clusters in the same area. Specifically, the diameter histogram (see left panel of Figure 1c) shows a narrow size distribution with a mean cluster size about 0.40±0.03 nm, a value agreeing very well with the expected value for the 3D Cu_5_. This estimation of the clusters size is close to that previously reported for similar samples.^[1]^ A small proportion (≈<10–15 %) of smaller Cu_3_ (not detected in the luminescence spectra) could also be present in the samples.

**Figure 2:**
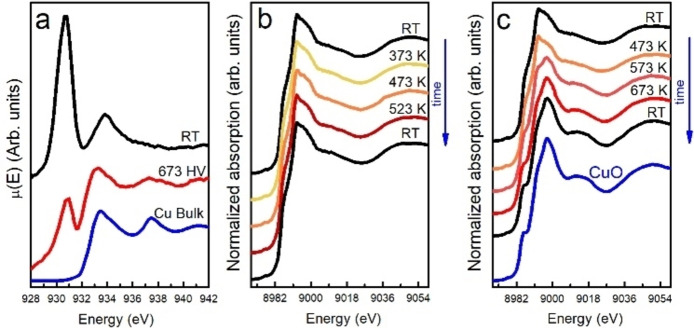
a, Cu L_3_‐edge XANES spectra of Cu_5_/HOPG in high concentration. Spectra were collected in high vacuum at RT (black) and after at 673 K (red). The spectrum of metallic Cu is shown for comparison (blue). b, XANES spectra at the Cu K‐edge of Cu_5_/HOPG with the same concentration as in a, collected in air from RT to 523 K and back to RT. c, XANES spectra at the Cu K‐edge of the same sample used in b, collected in air from RT up to 673 K and back to RT. The spectrum of CuO reference (blue) is shown for comparison. Each spectrum that is presented corresponds to a condition that was reached after waiting for the spectrum to not change.

### In situ XANES and NAP‐XPS Experiments

To investigate the stability of Cu_5_ under different conditions, we performed XANES experiments at the Cu L_3_ and K‐edges at different temperatures in high vacuum (HV) (about 1.5×10^−7^ mbar), in a low O_2_ pressure and in air. Cu_5_ were deposited on HOPG (Cu_5_/HOPG) (see Supporting Information), which exhibits a weak interaction with the clusters.^[10]^ According to an experimental estimation by XPS, considering the intensity of Cu 1s and Cu 2*p* photopeaks (see Supporting Information Figure 3), the final concentration of Cu in the sample for these experiments represents about ten monolayers of Cu_5_ on HOPG. Figure 2a shows the XANES spectra at the Cu L_3_‐edge of Cu_5_/HOPG when heated from room temperature (RT) up to 673 K in HV. From the spectrum taken at RT in HV we can conclude that Cu atoms in the clusters present a mixture of oxidation states with a very small proportion of Cu(II). After heating at 673 K in HV, bulk metallic Cu is formed predominantly, as is evidenced by the appearance of the two bumps at 937 eV and 941 eV in the red spectrum in Figure 2a. The XANES spectrum did not change when going back to RT so this was an irreversible transformation. Figure 2b shows the Cu K‐edge XANES spectra of the clusters while heating from RT to 523 K and cooling down back to RT in air at atmospheric pressure. Prior to XANES characterization, samples were treated in order to remove the hydration shell from the mother's solution (see Supporting Information Figures 4 and 5). All spectra exhibit the characteristics associated with Cu(II), i. e., an energy edge located at 8986 eV and a weak feature (pre‐peak) at 8977 eV. When the sample is cooled down to RT in air no changes in the spectra are observed showing that Cu_5_ are structurally and chemically stable up to 523 K in air. However, when the temperature is increased above 573 K, Cu_5_ lose their stability (Figure 2c). Indeed, XANES spectrum shows drastic changes at 673 K, with no modifications when cooled to RT, indicating an irreversible transformation. The final XANES spectrum obtained corresponds to that of bulk CuO. Both experiments in HV and air suggests the coalescence of the Cu_5_ when heated to 673 K, leading to the formation of bigger copper domains, resulting in the appearance of metallic Cu in HV and CuO in air, resembling the behavior of Cu nanoparticles.

**Figure 3:**
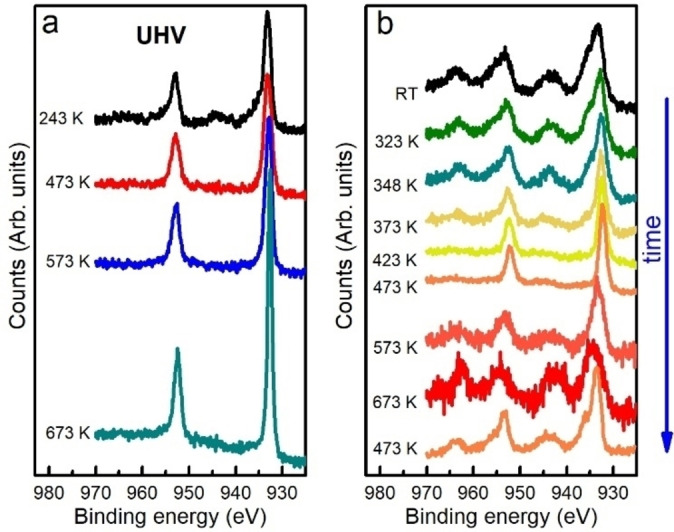
Cu 2*p* XPS spectra of Cu_5_/HOPG collected with a photon energy of 1350 eV. a, Spectra taken at the indicated temperatures in HV. b) Spectra taken in 0.15 mbar of O_2_ from RT to 673 K and back to 473 K. Each spectrum that is presented corresponds to a condition that was reached after waiting for the spectrum to not change.

**Figure 4:**
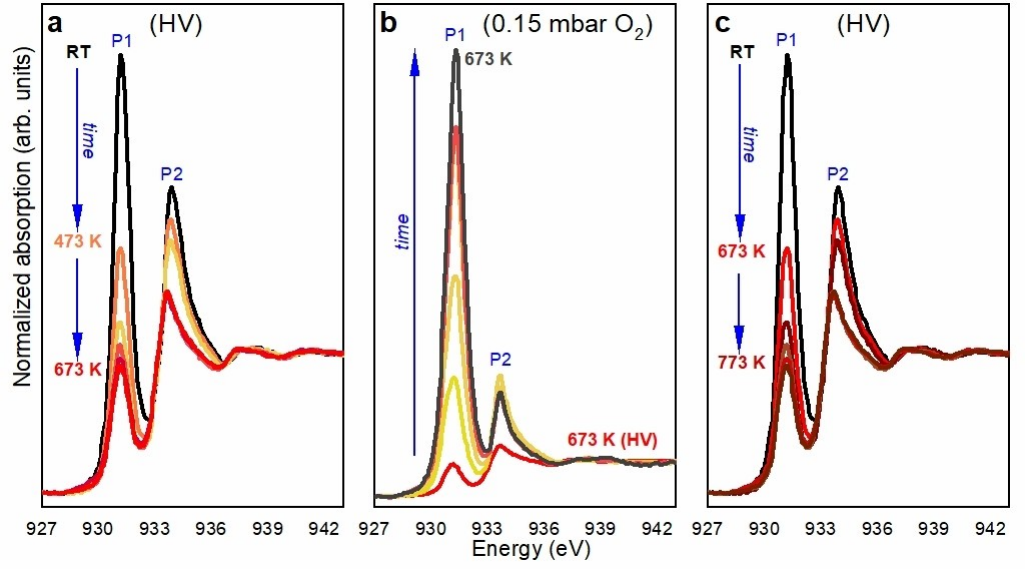
In‐situ Cu L_3_‐edge XANES spectra of Cu_5_/HOPG in low concentration (see text). a, Stage 1, in HV during heating. b, Stage 2: 0.15 mbar of oxygen pressure at 673 K. c, Stage 3: reduction during heating in HV. Each spectrum that is presented corresponds to a condition that was reached after waiting for the spectrum to not change.

**Figure 5:**
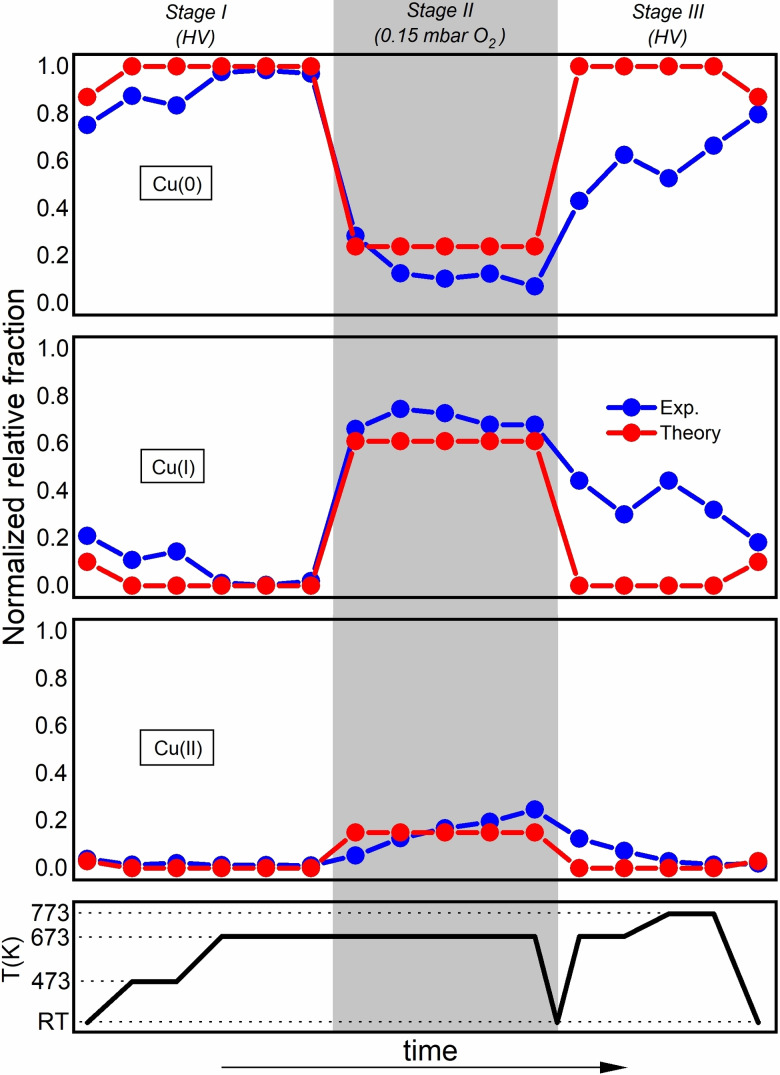
Comparison between theoretically and experimentally determined fractions of Cu(0), Cu(I) and Cu(II) oxidation states in Cu_5_/HOPG in HV and 0.15 mbar of O_2_. Theoretical values are determined through a Boltzmann‐weighted average of the Helmholtz free energies for each complex and their associated distributions of oxidation states (see Supporting Information Section 9). Error bars of experimental points are not shown for simplicity. The time axis is only indicative of the experimental sequence for each condition. Each spectrum used to obtain this figure corresponds to a condition that was reached after waiting for the spectrum to not change.

NAP‐XPS experiments employing the same Cu_5_ concentration on HOPG (i. e. about ten monolayers) as in the XANES experiments were performed. Different oxygen pressures were examined to estimate the attenuation of the signal by the gas phase (see Supporting Information Figure 6). 0.15 mbar of O_2_ was chosen as the working pressure.

**Figure 6:**
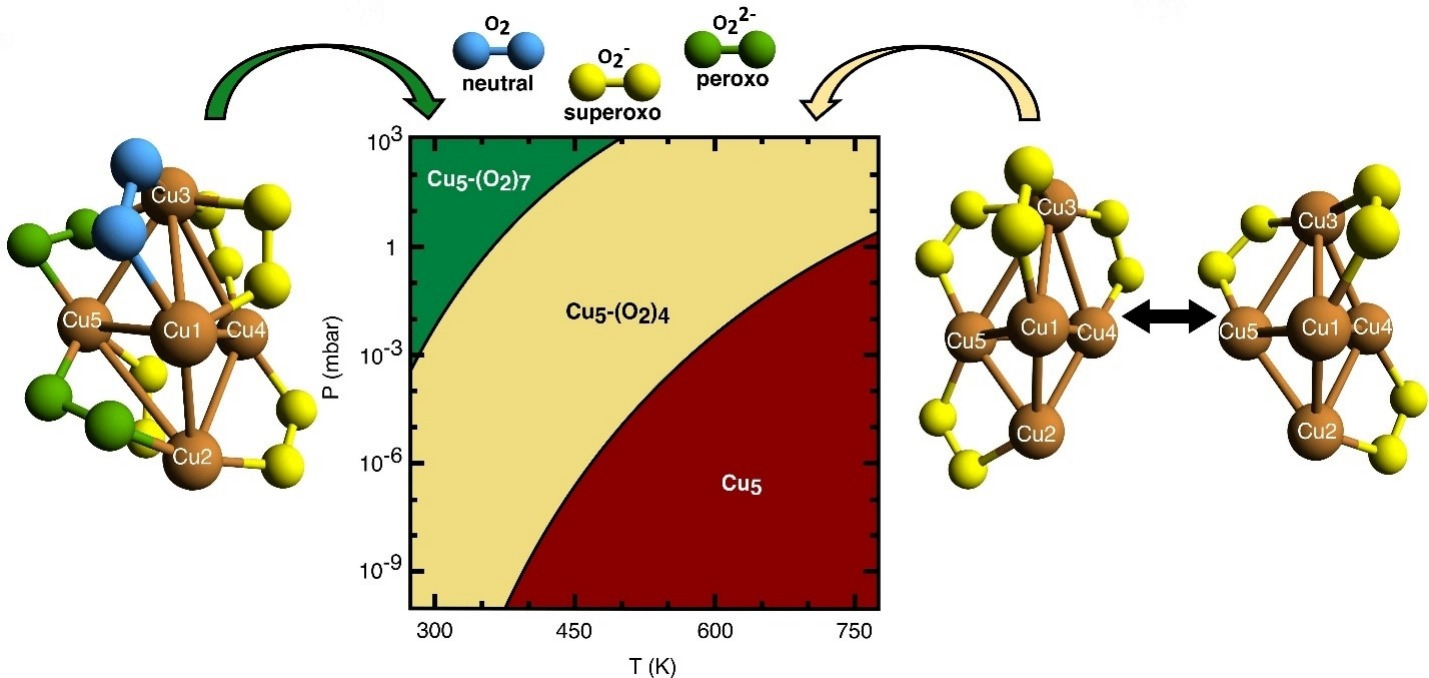
Theoretical characterization of Cu_5_‐(O_2_)_n_ complexes. Phase diagram, showing the most probable Cu_5_−(O_2_)_n_ complexes at each variable pair (p , T ). Their optimized structures are also presented (at T =0 K).

XPS allows a qualitative identification of Cu(0), Cu(I) and Cu(II) oxidation states. XPS spectrum of Cu(II) present broad satellite features at 943 eV and 963 eV while 2*p*_*3/2*_ and 2*p*_*1/2*_ photopeaks become shifted by 0.5 eV, and are significantly broader than those observed in Cu(I) and Cu(0) spectra. The Cu(I) state can only be differentiated from Cu(0) by a satellite peak at 945 eV.

Figure 3 shows the Cu 2*p* photoemission peaks of the Cu_5_/HOPG, with the same concentration used for XANES experiments, but measured from RT to 673 K in HV (Figure 3a) and in 0.15 mbar of O_2_ (Figure 3b). Under HV conditions only Cu atoms with Cu(I) oxidation state were observed below 423 K and they are reduced as temperature was increased. The narrowing and the increase of the intensity of the photoemission peaks above 573 K indicate the formation of agglomerated metallic Cu phases, as previously observed by XANES (Figure 2a). In the presence of O_2_ and below 373 K, the shape of the photopeaks and the appearance of the satellites peaks indicate that the main state of Cu atoms is Cu(II). An unexpected behavior was observed between 373 and 473 K, because a drastic reduction of the Cu atoms oxidation state occurred, indicated by the narrowing of the photoemission peaks and the disappearance of the satellite peaks. Above 573 K, photoemission peaks became broader and satellites reappeared indicating that the oxidation state of Cu atoms in the clusters is Cu(II) again. A lower oxidation state of Cu atoms was never recovered after heating at 673 K, as it is demonstrated in the final XPS spectrum taken at 473 K at the end of the treatment, which shows the presence of Cu(II) mainly (lowest spectrum in Figure 2c). This is consistent with an irreversible oxidation of the sample, as was observed in the XANES experiments, when the sample was heated up to 673 K in air. In summary, above a threshold temperature of 573–673 K, the Cu atoms in the Cu_5_/HOPG form bulk species. When the sample is in HV, metallic Cu is obtained, while in O_2_, even at low pressures as 0.15 mbar, CuO oxide is irreversibly formed.

It is important to highlight the particular behavior observed between 373 K and 473 K. When heating a Cu surface in the presence of oxygen, one would expect to observe a signal corresponding to oxidized Cu. In our case, for Cu_5_/HOPG heated from RT to 673 K, Cu(II) was clearly observed for all the sequence, except in the range from 373 K to 473 K, where Cu(I) and Cu(0) were detected. To our knowledge, this is the first experimental evidence for a reduction of Cu atoms in small structures by heating in O_2_. On the contrary, heating Cu surfaces and nanoparticles is known to drive the oxidation of Cu atoms as reported in the literature.^[27]^

### Monolayer of Cu clusters

To further investigate the resistance of isolated clusters to oxidation, we performed XANES experiments at the Cu L_3_‐edge with lower concentrations to form no more than one monolayer of Cu_5_ clusters on HOPG and prevent their coalescence. XANES at the Cu L_3_‐edge allows the quantification of copper oxide mixtures. CuO and Cu_2_O have strong absorption edges at 931.3 eV and 933.7 eV respectively, and substantial shape differences between them and the metallic Cu, allowing an easy identification of each oxidation state (see Supporting Information Figure S7). Thus, the XANES spectra of those compounds can be used as reliable fingerprints to identify the different oxidation states and their relative concentrations in the sample under different thermodynamic equilibrium conditions (referred to as “Stages” in this manuscript).

Figure 4 shows the in‐situ Cu L_3_‐edge XANES spectra of Cu_5_/HOPG. During the heating form RT to 673 K in HV (Stage 1), a pronounced decrease of the peak at 931.3 eV (P1) with the simultaneous increase of the one at 933.7 eV (P2) were observed (see Figure 4a), with no evidence of bulk metallic Cu formation (see Supporting Information Figure S7). After this initial treatment, 0.15 mbar of oxygen were introduced in the chamber while keeping the temperature at 673 K (see Figure 4b, Stage 2). During this stage, the intensity of the P1 signal present in the initial phase of Stage 1 was recovered and even exceeded with the decrease of the P2 intensity, indicating strong oxidation of Cu atoms in the clusters. Finally, after reaching the equilibrium, the oxygen leak valve was closed, and the sample was cooled to RT. After reaching that temperature, the sample was heated up to 773 K in HV (see Figure 4c, Stage 3).

A linear combination fitting analysis of the Cu L_3_‐edge XANES spectra was performed to quantify the percentage of the Cu species with different oxidation states. The spectra of metallic Cu, CuO and Cu_2_O were used as standards for the different oxidation states following the procedure described by Eren et al.^[28]^ (see Supporting Information Section 7 and Supporting Information Figure S8) as the energy positions of the 2*p*→3*d* transition peaks in the Cu L_2,3_ XANES spectra are strongly influenced by the chemical states of the Cu.^[29]^ In particular, the amount of unoccupied *d* orbital character, and hence, the strength of the sharp XANES peak at about 931 eV, is related to the amount of Cu(II). Complementary, the spectrum of monovalent Cu (formally Cu(I)) do not show any peak at 931 eV because the *d* shell is essentially full.

Figure 5 shows the fractions of oxidation states of Cu atoms in Cu_5_/HOPG as a function of temperature and O_2_ pressure. The initial fractions of Cu species are recovered after the complete cycle showing full reversibility in the oxidation/reduction process, pointing out that some reversible mechanisms (as the molecular oxygen adsorption/desorption which we will describe below) should be involved. In order to confirm these results a new sample of Cu_5_/HOPG with even lower concentration (less than one monolayer) was prepared and characterized by Cu L_3_‐edge XANES in 0.15 mbar of O_2_ and different temperatures (see Supporting Information Figure S9). The results are identical to those obtained with one monolayer and confirm the reversible behavior of the clusters in their interaction with O_2_.

### Theoretical Modelling: Molecular Oxidation

To explain the observed reversible oxidation, we assume that Cu_5_ can form a complex with several adsorbed O_2_ molecules. This way, the experimentally determined relative concentration of oxidation states (SI Figure S9) can be reproduced theoretically by calculating the number of O_2_ molecules that can be adsorbed depending on the oxygen pressure and temperature (Figure 6). As theoretically assessed in Refs. [23], [30], we further take into account the fact that the energy barriers from physisorption to chemisorption states are very low (ca. 0.1 eV in Ref. [23]) also in the case of the co‐adsorption of multiple O_2_ molecules, whereas those associated to O_2_ bond breaking for superoxo ($O_{2}^{-}$ ) species remain very high (>4 eV in Refs. [23,30]). Thus, although the formation of reaction products involving dissociated O_2_ molecules can be thermodynamically favorable, it would be kinetically forbidden at the experimental temperature range from RT up to 773 K. Therefore, a realistic model should account for that: 1) the energy barriers for O−O bond breaking are extremely high, hindering an irreversible oxidation, and 2) low energy barriers from physisorption to chemisorption states, favoring reversible molecular oxidation processes upon successive steps of attachment/release of O_2_ molecules. Once assured that O_2_ adsorbs to Cu_5_ in molecular form, dispersion‐corrected DFT is employed to obtain optimized geometries of large molecular Cu_5_‐(O_2_)_n_ complexes (n≤10). The structural optimizations are carried out using the Perdew‐Burke‐Ernzerhof (PBE) density functional^[31]^ and the Becke‐Johnson (BJ) damping^[32]^ for the D3 dispersion correction. The optimized Cu_5_−(O_2_)_n_ structures (Figure 6 and Supporting Information Figure S11) show enhanced stability when the O_2_ molecules attach to bridge Cu_5_ positions. Depending on their number, the O_2_ molecules can be absorbed as neutral (O_2_), superoxo ($O_{2}^{-}$ ), or peroxo ($O_{2}^{2-}$ ) species. Since dispersion forces allow a stretching of the Cu−Cu distances, the Cu_5_ can adapt its shape to accommodate the charged O_2_ species, featuring larger O−O bonds, at its bridge sites (see Supporting Information Figure S13).

The adsorption of (up to 7, mostly charged) O_2_ molecules on Cu_5_ is the result of a charge‐transfer process where all Cu atoms collectively participate in the donation of electronic charge to the O_2_ molecules, leading to an increase of the Cu−Cu distances. A “breathing” effect due to concerted elongations/contractions of the Cu−Cu bonds enables the adsorption/release of O_2_ molecules (see Supporting Information Figure S14). As shown in the same figure, the negative charge is collectively donated from the 3*d* orbitals of the copper atoms and shared by the π^✶^ orbitals of several O_2_ molecules. The charge donation from one copper atom to one O_2_ molecule activates a reorganization of the subnanometer‐sized network formed by the 3*d* orbitals of all copper atoms, making a collective charge donation possible, also illustrated in Supporting Information Figures S14 and S15. A collective back‐donation transfer process from *s*‐type orbitals of the adsorbed O_2_ molecules to *p*‐type orbitals of the copper atoms has been also identified (see Supporting Information Figure S15). The collective adsorption process thus leads to the migration of electron charge from the Cu clusters to the adsorbed O_2_ molecules on its surface but not to the rupture of Cu−Cu and O−O chemical bonds, making it possible for the clusters to recover their metallic phase upon O_2_ release by heating or decreasing the oxygen pressure, as experimentally observed and quantitatively reproduced by theory, as we will describe below.

### Theoretical Phase Diagram of Cu_5_‐(O_2_)_n_ Complexes and Comparison with Experiments

At a given temperature (T ) and partial oxygen pressure (p ), we have determined the relative stability of complexes Cu_5_‐(O_2_)_n_ by calculating the thermodynamical potential ω [^30^, ^31^, ^32^, ^33^, ^34^, ^35^]

(1) $$\omega \left(T,\mu_{O_{2}},n\right)=\;\Delta E_{F,corr}\left(T\right)-T\cdot s_{{Cu}_{5}-(O_{2}{)}_{n}}\left(T\right)+T\cdot s_{{Cu}_{5}}\left(T\right)-n\cdot {\bar{\mu }}_{O_{2}}\left(p,T\right)$$

This way (see Supporting Information Section S11 for details), the Cu_5_ are treated as fully immobilized on the support and coupled to a heat bath of temperature T and an infinite reservoir of O_2_ gas at pressure p . Under these idealized conditions, the ω potential will become minimal at thermodynamic equilibrium. From this expression, the number *n* of adsorbed O_2_ molecules which minimizes ω for a specified temperature and a given oxygen pressure can be obtained as follows: $\Delta E_{F,corr}$ , the first term on the right‐hand side of Equation (1), corresponds to the formation energy of Cu_5_‐(O_2_)_n_ and is defined as

(2) $$\Delta E_{F,corr}\left(T\right)=E_{{Cu_{5}-\left(O_{2}\right)}_{n}}-E_{Cu_{5}}-n\cdot E_{O_{2}}+E_{corr}\left(T,n\right)$$

where $E_{{Cu_{5}-\left(O_{2}\right)}_{n}}$ and $E_{Cu_{5}}$ denote the DFT energies of the oxygen‐covered and pure cluster, respectively, and $E_{O_{2}}$ is the DFT energy of molecular oxygen. $E_{corr}$ is introduced to correct the internal energy contribution with the zero‐point energy, the thermal vibrational contribution as well as thermal rotational, and translational terms. The next term on the righthand side of Equation (1) introduces a correction with respect to the entropy $s_{{Cu}_{5}-(O_{2}{)}_{n}}$ of the cluster. The last term on the right side of Equation (1) is the temperature and pressure dependent part of the chemical potential of molecular oxygen expressed as,

(3) $${\bar{\mu }}_{O_{2}}\left(p,T\right)=\Delta h_{O_{2}}\left(p_{0},T\right)-T\cdot s_{O_{2}}\left(p_{0},T\right)+R\cdot T\;ln\left(\frac{p}{p_{0}}\right)$$

where the pressure enters through the ratio $p/p_{0}$ with the reference oxygen pressure $p_{0}$ set to 1 atm (ca. 1013 mbar). Note that $\mu_{O_{2}}\left(p,T\right)=E_{O_{2}}+{\bar{\mu }}_{O_{2}}\left(p,T\right)$ , but the T , p ‐independent contribution to the O_2_ chemical potential, $E_{O_{2}}$ , has been moved to the $\Delta E_{F,corr}\left(T\right)$ term (Eq. (2)). This is a convenient way of separating the overall free energy change into pressure‐independent and pressure‐dependent contributions. The change of enthalpy is given by $\Delta h_{O_{2}}=h\left(p_{0},T\right)-h\left(p_{0},T=0\;K\right)$ . For maximum accuracy, values for $h_{O_{2}}$ and $s_{O_{2}}$ taken from the NIST database are used.[^36^, ^37^]

The (p , T )‐phase diagram is created by determining the number n of adsorbed O_2_ molecules which minimizes ω for a specified temperature and a given oxygen pressure (see Figure 6 and Supporting Information Figure S17). Measurable oxidation states [Cu(0), Cu(I), Cu(II)] can be assigned to each copper atom for a given Cu_5_−(O_2_)_n_ complex (see, for example, Supporting Information Figures S11 and S16). To fully account for the thermodynamical conditions, a Boltzmann‐weighted average of their Helmholtz free energies and associated distributions of oxidation states is carried out for each variable pair (p , T ) (see Figure 6 and Supporting Information Table S2).

At RT and atmospheric pressure, the Cu_5_−(O_2_)_7_ complex happens to be the most stable, with $\Delta F_{f}$ < −5 eV (see Supporting Information Figure S17). All oxygen molecules become adsorbed at bridge sites of the Cu_5_ clusters as neutral (O_2_), superoxo ($O_{2}^{-}$ ), or peroxo ($O_{2}^{2-}$ ) species, with most of the copper atoms bearing the Cu(II) oxidation state. In fact, as can be seen in Figure 6, the phase of the Cu_5_−(O_2_)_7_ complex (shown in green) persists up to about 500 K at atmospheric pressure. The analysis of the wave‐function obtained using multi‐reference theory for the Cu_5_−(O_2_)_7_ complex (see Supporting Information Section 10) reveals a spin density close to unity for most Cu atoms, a clear signature of Cu(II) oxidation states.

At atmospheric pressure, upon heating to ca. 500 K, the Cu_5_−(O_2_)_7_ complex loses O_2_ molecules, and the Cu_5_−(O_2_)_4_ and Cu_5_−(O_2_)_3_ complexes become the most stable, with a free energy of about −4 eV (see Supporting Information Figure S17). When the pressure is lowered to ca. 0.15 mbar at 350 K, the Cu_5_−(O_2_)_4_ complex is the most stable (see Figure 6), being quasi‐iso‐energetic with the Cu_5_−(O_2_)_3_ complex, another feature reflecting the structural fluxionality of sub‐nanometric clusters. The analysis of the wave‐function obtained using multireference theory confirms that the Cu_5_ clusters become carriers of superoxo $O_{2}^{-}$ radicals (with a spin very close to unity), with most of the copper atoms assigned to the Cu(I) oxidation state (see Supporting Information Section 10.1 and Figure S17). These complexes are still stable upon heating to 673 K, explaining why the experiment shows that the Cu(I) oxidation state is dominant (see Figure 5). Further lowering of the oxygen pressure from 0.15 mbar to HV at a constant temperature of 673 K makes the copper cluster lose all O_2_ molecules so that the bare Cu_5_ cluster appear in the phase diagram as the predominant species (red area in Figure 6). This outcome clearly signals the occurrence of a reversible molecular oxidation, with the Cu_5_ clusters recovering the donated charge upon O_2_ desorption. Consequently, the Cu(0) oxidation state becomes the major component, as experimentally shown in HV and 673 K (Figure 5).

Reactivity is expected to be under kinetic control at RT and HV and the O_2_ molecules can become trapped at the physisorption minimum since there is a low, yet noticeable barrier between physisorption and molecular chemisorption states (ca. 0.1 eV, see Ref. [23]). Once the probability of trapping at the physisorption state is considered, the theoretical model predicts the Cu(0) oxidation state to be dominant (see Figure 5). Using the Boltzmann‐weighted average of the free energies for all complexes in their corresponding oxidation states, we reach a clear quantitative agreement with the experimentally determined fractions at 0.15 mbar as well (see Figure 5).

## Conclusions

Bare Cu clusters of five atoms, synthesized by an improved version of a previously developed electrochemical method show an outstanding stability against oxidation on HOPG, at least up to 773 K in 0.15 mbar of oxygen pressure. Cu_5_ clusters display a reversible O_2_ adsorption behavior, circling through different Cu oxidation states at varying temperature and oxygen pressure. The experimental results reveal a different behavior of Cu_5_ from the usually observed for bulk Cu or Cu nanomaterials. For the latter, increasing temperature favors their irreversible oxidation whereas for the presently investigated clusters, it favors the desorption of oxygen, leaving the clusters in an unoxidized state. However, even being resistant to oxidation, clusters are not immune to coalescence and consequent formation of nanoparticles. The temperature at which this process occurs depends on the concentration, being about 573 K when they are deposited in multilayers, and higher than 773 K when deposition does not exceed one monolayer. These results are important for the application of clusters in catalysis, firstly with respect to reversible oxygen adsorption, and secondly, with respect to the adequate choice of concentrations and supports, in order to prevent agglomerations on the surface to minimize surface diffusion.

As demonstrated by applying multireference ab initio theory to the case of a single O_2_ molecule in Ref. [23], this noble‐like behavior is favored by both the low values of the energetic barriers (ca. 0.1 eV) from physisorption to molecular O_2_ chemisorption states as well as the high O_2_ dissociation energy barriers (>4 eV). Combining dispersion‐corrected DFT theory with first principles thermochemistry, a phase diagram of Cu_5_−(O_2_)_n_ complexes (n≤10) is created, matching the experimental observations performed during reaction conditions. The reversible adsorption of O_2_ is the result of concerted rearrangements of the atomic nuclei and coordinated charge transfer processes within a network of Cu 3*d* orbitals. Our findings contribute to the understanding of the fundamental mechanisms driving clusters oxidation/reduction processes. According to the proposed model, the disclosed collective mechanism has its origin in the sub‐nanometer size of the actual quantum system and, particularly, on its ability to exhibit wide amplitude atomic nuclei motion. The direct evidence on the air‐stability of Cu_5_ clusters here obtained opens a way for applications at the energy and environmental technologies, such as in visible light photo‐catalysis.[^16^, ^38^, ^39^]

## Experimental Section

**Materials and Methods**: Copper clusters were obtained by using an electrochemical method with an Autolab PGSTAT 20 potentiostat. A Methrom thermostated‐3 electrode electrochemical cell was employed, with a copper sheet of 10 cm^2^ as the working electrode, a platinum sheet of 10 cm^2^ as the counter electrode, and a hydrogen electrode as the reference. The working and counter electrodes were placed vertically face to face at a distance of 1.5 cm. Pure MilliQ water (conductivity ≈6.26 μΩ/cm^3^) without any added electrolyte was used, and N_2_ was bubbled during 30 min in order to deareate the solution. The synthesis was carried out at constant temperature (298 K) at a constant Voltage of 1 V for 1500 s. The Cu sheets were carefully cleaned before the synthesis: they were first polished with sandpaper (600 grid) followed by alumina (≈50 nm), washed out thoroughly with MilliQ water and sonicated. After the synthesis, the remaining Cu^2+^ ions were precipitated by NaOH (pH≈12), subsequent filtration, and finally the pH was adjusted to 7 by addition of HClO_4_. A typical concentration of clusters obtained after purification is in the range ≈40 mg/L. The typical yield of cluster synthesis, considering the difference between Cu content obtained by flame atomic absorption spectroscopy and the Cu^2+^ content obtained by ion selective electrode, is around 70 %. HOPG supported Cu_5_ clusters were prepared by a simple dripping method. Water solution containing approximately 100 mg/L (i. e.,100 μg/mL) of Cu_5_ clusters was dropped onto highly oriented pyrolytic graphite (NT‐MDTZYB 10×10×2.0 mm) avoiding the contact of the solution with the HOPG borders. HOPG was previously cleaned by several mechanical exfoliations using the sticky tape method. After deposition, the HOPG surface was cleaned with MilliQ water to obtain a thin layer of Cu_5_ clusters and remove other impurities. The sample was then dried in air at 343 K for 1 h. Samples for electron microscopy studies were prepared by depositing one drop of the synthesized clusters solution (1 : 10000 diluted, i. e., with a cluster concentration ≈10 ng/mL, which would correspond to less than 1 monolayer of clusters) onto holey‐carbon coated Au grids. After their preparation, the TEM samples were conserved under vacuum conditions.

Scanning‐Transmission Electron Microscopy studies, using High‐Angle Annular Dark‐Field, HAADF‐STEM, which contrasts are related to the roughly Z2 atomic number of the elements under the beam, were performed on a FEI Titan Themis 60−300 Double Aberration Corrected microscope operated at 200 kV. We corrected the aberrations of the condenser lenses up to fourth order, using the Zemlin tableau to obtain a sub‐Angstrom electron probe. A condenser aperture of 50 μm yielding an electron probe with a convergence angle of 20 mrad was used. To limit the damage by the electron beam, a fast image recording protocol was used by combining a beam current of 25 pA, a 2.5 μs dwell time and an automated finetuning alignment of A1 and C1 using the OptiSTEM software. To obtain images with good quality, the beam current and image acquisition time should be optimized according to the stability of the sample under the beam. Aimed to quantitatively characterize the Cu clusters, a specific methodology for the digital analysis of the experimental images has been developed and coded in a home‐made MATLAB script. First, to improve the signal‐to‐noise, the AC HAADF‐STEM images were denoised by combining the Anscombe variance stabilization transform (Anscombe VST) with the Undecimated Wavelet Transform (UWT). The background from the denoised images was subtracted by disk top‐hat filtering, allowing us to improve the visibility of the sub‐nanometric clusters.

XANES experiments at the Cu K‐edge were performed at the XAFS2 beamline^[40]^ of the Laboratorio Nacional de Luz Sincrotron (LNLS), Campinas, Brazil. The measurements were performed in fluorescence mode using a Si(111) crystal monochromator with a ion chamber as I_0_ detector and a Germanium 15 elements fluorescence detector, from Canberra Inc. The XANES spectra of a Cu foil and reference compounds were measured in transmission mode using two ion chambers as detector. The X‐ray Absorption spectra were normalized by standard methods using the ATHENA software which is part of the IFFEFIT package^[41]^ in order to obtained the normalized XANES spectra.

The NAP‐XPS experiments were carried out at the CIRCE beamline of the ALBA Synchrotron Light Source.^[42]^ The acquisition was performed using a PHOIBOS 150 NAP electron energy analyzer (SPECS GmbH) equipped with four differential pumping stages and a set of electrostatic lenses which enable the performance of XPS measurements with the sample at pressures from ultrahigh vacuum (UHV, with a base pressure of 10^−10^ mbar) up to 20 mbar. Most of the experiments were performed at a chamber pressure of 1.5×10^−7^ mbar, which we call High Vacuum (HV). All NAP‐XPS measurements have been acquired with 1350 eV photon energy. XANES at the Cu‐L_3_ edge were also performed at the same end station measured by Total Electron Yield (TEY). The current from the sample was amplified with the ALBA Em current amplifier and was normalized to the incident photon flux, measured via the Au‐coated refocusing mirror. The spot size for both NAP‐XPS and XANES measurements was ∼100×100 μm^2^. XANES techniques at the soft X‐ray region are usually referred as Near Edge X‐ray Absorption Fine Structure (NEXAFS), but we will maintain the XANES nomenclature to save acronyms that refer to the same physical phenomena.

**Computational Methods**: In all calculations on bare Cu_5_ clusters, a trigonal bipyramidal (3D) structure is assumed. Although HOPG‐supported Cu_5_ clusters are used in the experiment, it has been previously shown^[16]^ that these clusters are minimally perturbed by a carbon‐based surface (graphene) due to the dispersion‐dominated nature of the Cu_5_‐graphene interaction. Our theoretical approach combines density functional theory (DFT) and multireference perturbation theory.^[43]^ Due to the open‐shell nature of the interacting species, the application of the multireference method has allowed to ensure the nature of the oxidation states of the copper atoms in Cu_5_−(O_2_)_n_ complexes. The geometry optimization of Cu_5_−(O_2_)_n_ clusters geometries was performed at PBE−D3 level[^31^, ^32^, ^44^] given its excellent performance in describing supported and unsupported sub‐nanometer silver[^45^, ^46^] and copper[^5^, ^24^] clusters. A reoptimization of selected structure using the D4 Grimme's parameterization^[47]^ modifies the Cu−Cu bond length by less than 10^−3^ Å. We used the atom‐centred def2‐TZVP^[48]^ basis set for copper and oxygen atoms. The Helmholtz free energies of formation were calculated using the def2‐QZVPP basis set at the relaxed geometries, counterpoise‐corrected, with the frequencies calculated with the def2‐ TZVP basis set. These calculations were realized with the ORCA^[49]^ suite of programs (version 4.0.1.2). The chemical oxidation states of the copper atoms for each Cu_5_−(O_2_)_n_ complex were deduced from an analysis of Mulliken charges^[50]^ and atomic spin populations with the Hirshfeld method.[^51^, ^52^] In order to assess the nature of the oxidation states of the Cu atoms and the neutral/peroxo/superoxol character of the adsorbed O_2_ molecules in Cu_5_−(O_2_)_n_ complexes, we carried out single‐state CASSCF calculations, using the most recent version of the MOLPRO code.^[53]^ We used the polarized correlation‐consistent triple‐ζ basis of Dunning and collaborators^[54]^ (cc‐pVTZ) for oxygen atoms, and the cc‐pVTZ‐PP basis set for copper atoms^[55]^ including a small (10‐valence‐electron) relativistic pseudopotential.

## Supporting Information

Additional references cited within the Supporting Information.[^56^, ^57^, ^58^, ^59^, ^60^, ^61^, ^62^, ^63^, ^64^, ^65^, ^66^, ^67^, ^68^, ^69^, ^70^, ^71^, ^72^, ^73^, ^74^, ^75^, ^76^, ^77^, ^78^, ^79^, ^80^, ^81^, ^82^, ^83^, ^84^, ^85^, ^86^, ^87^, ^88^, ^89^]

## Conflict of interest

The authors declare no conflict of interest.

1

## Supporting information

As a service to our authors and readers, this journal provides supporting information supplied by the authors. Such materials are peer reviewed and may be re‐organized for online delivery, but are not copy‐edited or typeset. Technical support issues arising from supporting information (other than missing files) should be addressed to the authors.

Supporting Information

## Data Availability

The data that support the findings of this study are available in the supplementary material of this article.
