# Supplementary material for: Stability and Reversible Oxidation of Sub‐Nanometric Cu5 Metal Clusters: Integrated Experimental Study and Theoretical Modeling
Source: Chemistry. 2023 Jul 7;29(49):e202301517. doi: 10.1002/chem.202301517 (PMC10946568; doi:10.1002/chem.202301517)
Supplement: Supplementary file 1 — Supporting Information [file CHEM-29-0-s001.pdf]

Chemistry–A European Journal

Supporting Information

Stability and Reversible Oxidation of Sub-Nanometric Cu_5 Metal Clusters: Integrated Experimental Study and Theoretical Modeling

David Buceta, Shahana Huseyinova, Miguel Cuerva, Héctor Lozano, Lisandro J. Giovanetti, José M. Ramallo-López, Patricia López-Caballero, Alexandre Zanchet, Alexander O. Mitrushchenkov, Andreas W. Hauser, Giampaolo Barone, Cristián Huck-Iriart, Carlos Escudero, Juan Carlos Hernández-Garrido, José Juan Calvino, Miguel López-Haro, María Pilar de Lara-Castells,* Félix G. Requejo,* and M. Arturo López-Quintela*

Contents

1 Figures **S2**

2 Tables **S19**

3 XPS quantification of Cu_5 clusters deposited on HOPG **S21**

4 Cu K-edge XANES experiments in air (high concentration of Cu_5 clusters) **S22**

5 Near Ambient Pressure XPS experiments at different oxygen pressures **S22**

6 Cu L_3-edge XANES experiments in high vacuum and in 0.15 mbar of oxygen (low concentration of Cu_5 clusters) **S23**

7 Methods used in the theory part **S24**

8 Molecular chemisorption states of Cu_5–$(O_2)_n$ complexes **S26**

9 Oxidation states of the copper atoms: multireference theory **S29**

9.1 Cu_5–$(O_2)_4$ S30

9.2 Cu_5–$(O_2)_7$ S32

10 Helmholtz free energies of formation and phase diagram of Cu_5–$(O_2)_n$ complexes **S33**

Supplementary Section 1: Figures

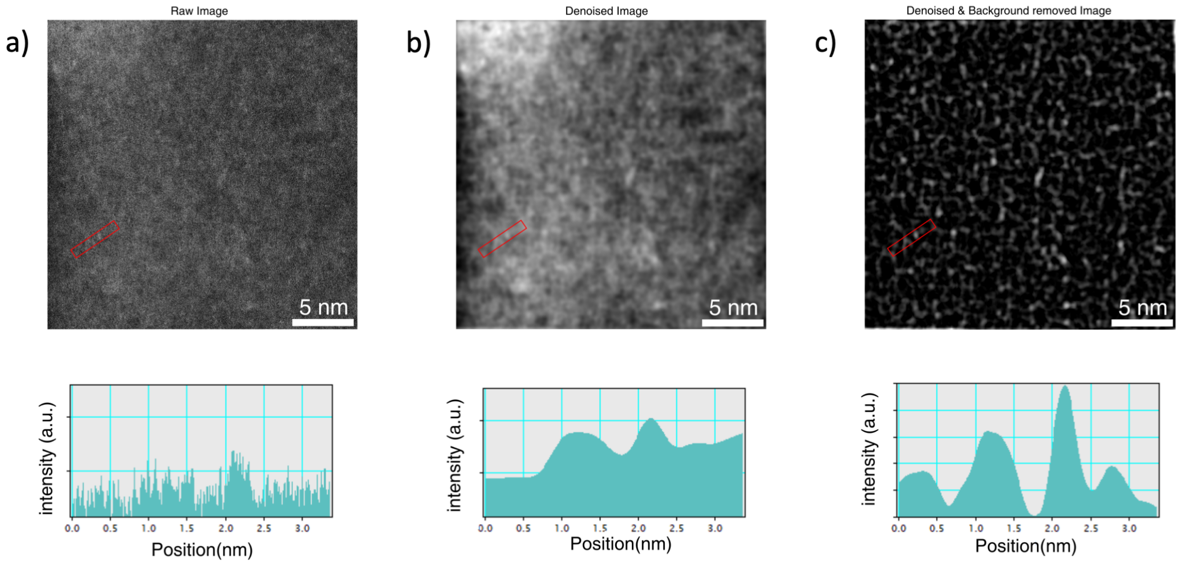

Supplementary Figure 1: **Schematic procedure for denoising of experimental HAADF-STEM images of the Cu_5 clusters. a,** Experimental AC HAADF-STEM image of the synthesized Cu_5 clusters. Intensity profiles, registered in a random area, illustrate the reduction of the noise within the background signals. **b,** Denoised image by combining Anscombe Variance Stabilization Transform (Anscombe VST) with the Undecimated Wavelet Transform (UWT). **c,** Background subtraction by disk tophat filtering.

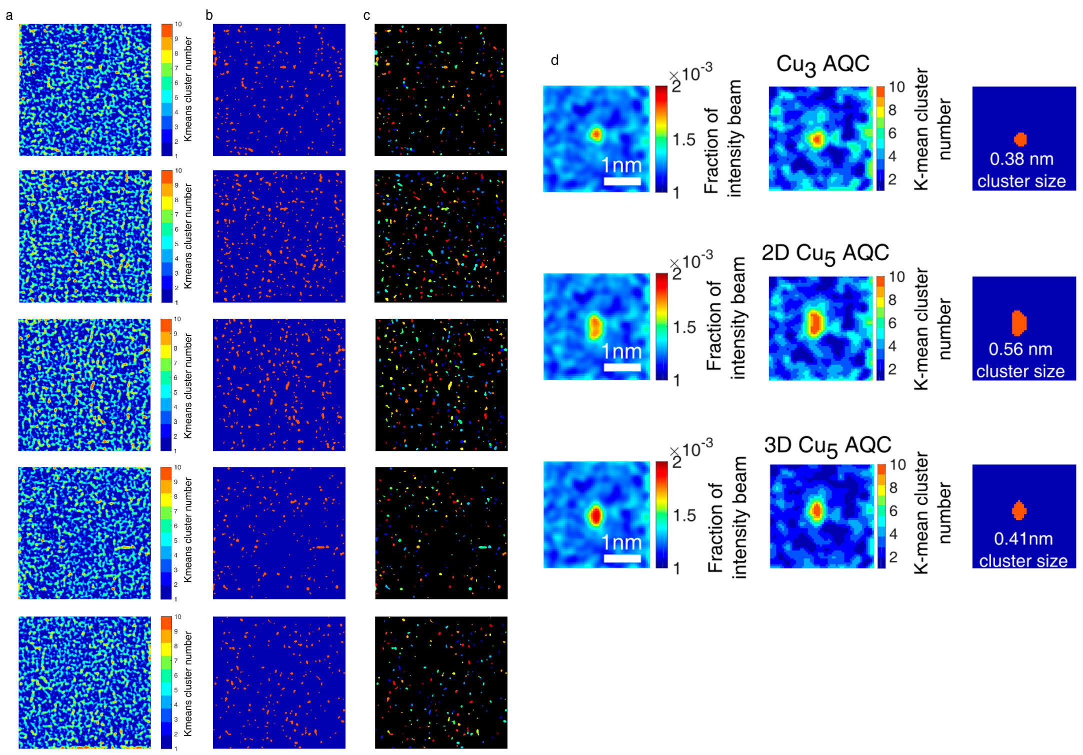

Supplementary Figure 2: **Analysis of AC HAADF-STEM images of Cu species by the *k*-means clustering method.** **a**, *k*-means clustering maps resulting from several raw images after denoising and background subtraction. **b**, Binarized images obtained from the *k*-means clustering analysis (Cu clusters in orange and background in blue). **c**, Pseudo-colour image after applying a watershed segmentation. Clusters with similar areas are displayed with the same colour. A total number of 1200 clusters were automatically detected and measured after clustering and segmentation. **d**, Characterization of modelled Cu clusters. Simulated HAADF-STEM images, after addition of Poisson and white Gaussian noise, from modelled Cu clusters with Cu_3-planar, Cu_5-planar (2D) and Cu_5-trigonal bipyramidal (3D) structures, respectively (left column). Results of the *k*-means cluster analysis from these modelled Cu_3-Cu_5 clusters and their segmentation by thresholding on binarized versions are also shown in the middle and right columns, respectively.

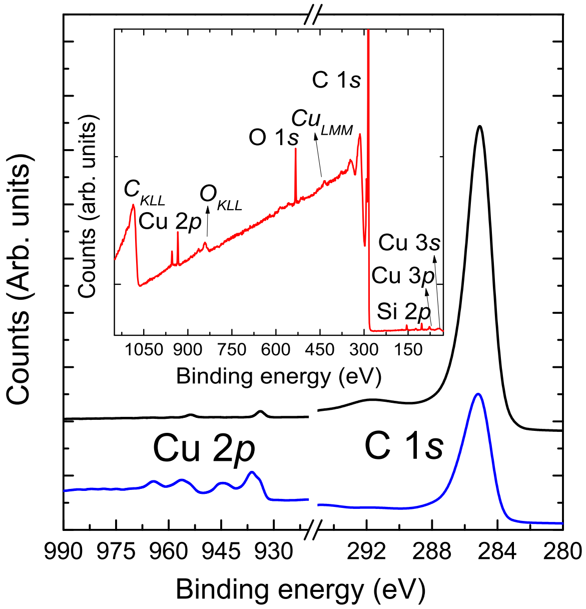

Supplementary Figure 3: **XPS spectra at the Cu 2*p* and C 1*s* levels for the "concentrated" and "monolayer" samples (shown with blue and black lines, respectively) taken from their respective surveys scans, as obtained in HV at RT with a incident photon energy of 1350 eV.** The x axis, indicating the binding energy, has been interrupted to better compare the regions associated to the Cu 2*p* and C 1*s* levels. The inset shows the complete XPS survey for the "monolayer" sample, where it is possible to notice the cleanliness of the sample. Only traces of Si are observed, which is a typical contaminant in HOPG substrates that do not interfere with the analysis carried out in this work.

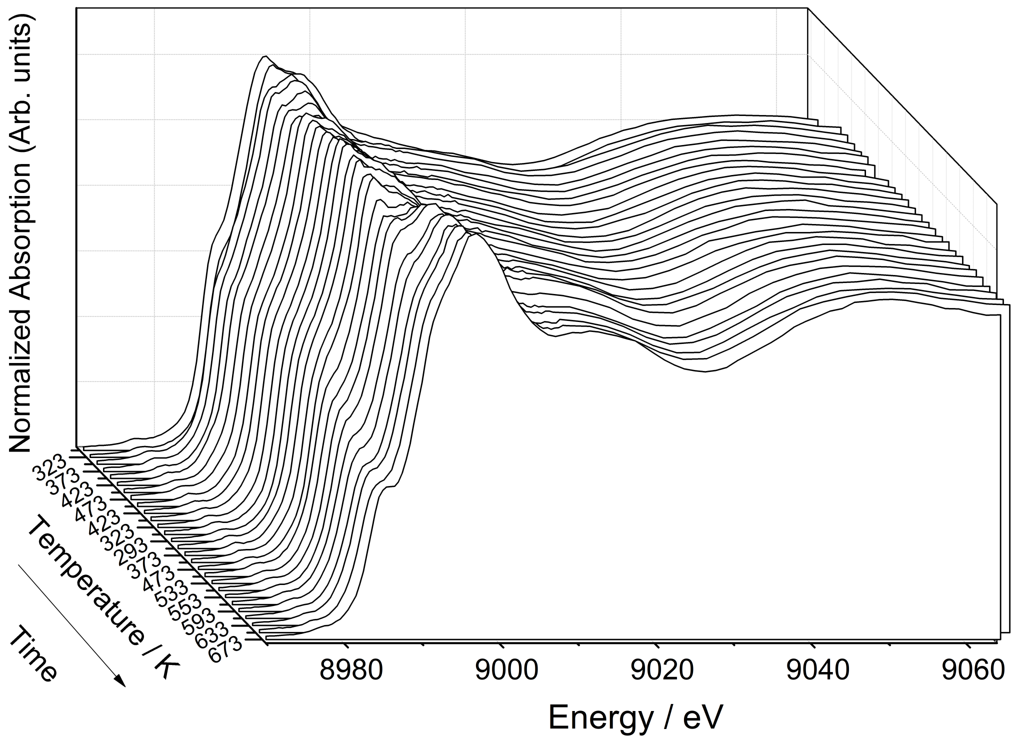

Supplementary Figure 4: **Cu K-edge XANES spectra of the Cu_5 clusters supported on HOPG obtained in air during the different cycles in the temperature range from RT to 673 K.**

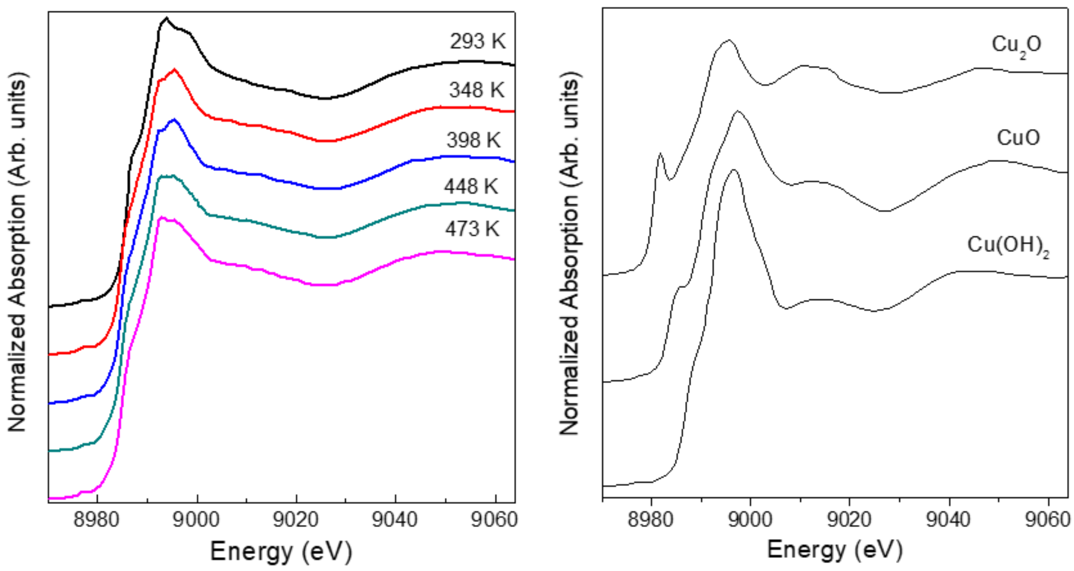

Supplementary Figure 5: **Left: XANES spectra at the Cu K-edge of the Cu_5 clusters supported on HOPG, as obtained in air from room temperature to 473 K during the first heating process. It is possible to observe slight differences between the spectra. Each spectrum that is presented corresponds to a condition that was reached after waiting for the spectrum to not change. Right: Spectra of Cu reference compounds**

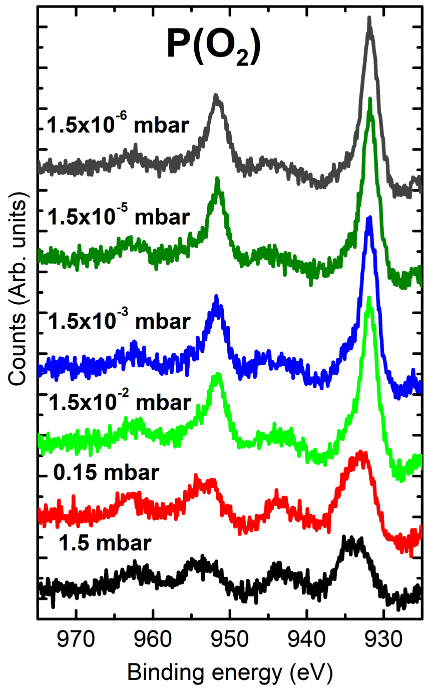

Supplementary Figure 6: **XPS spectra of Cu_5 clusters on HOPG for a sample with less than one monolayer of Cu_5 coverage at the Cu 2*p* photopeaks region.** All spectra were taken at room temperature at the indicated oxygen pressure.

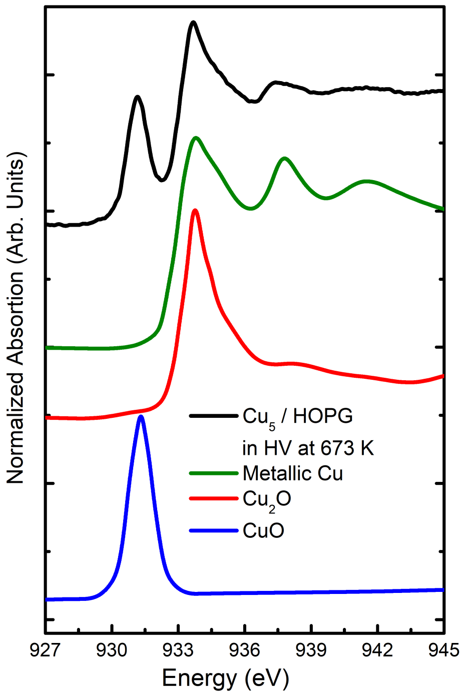

Supplementary Figure 7: **Cu L_3-edge XANES normalized spectra for metallic Cu, Cu_2O and CuO reference compounds together with that of Cu_5 clusters on HOPG sample in HV after heating in vacuum at 673 K.**

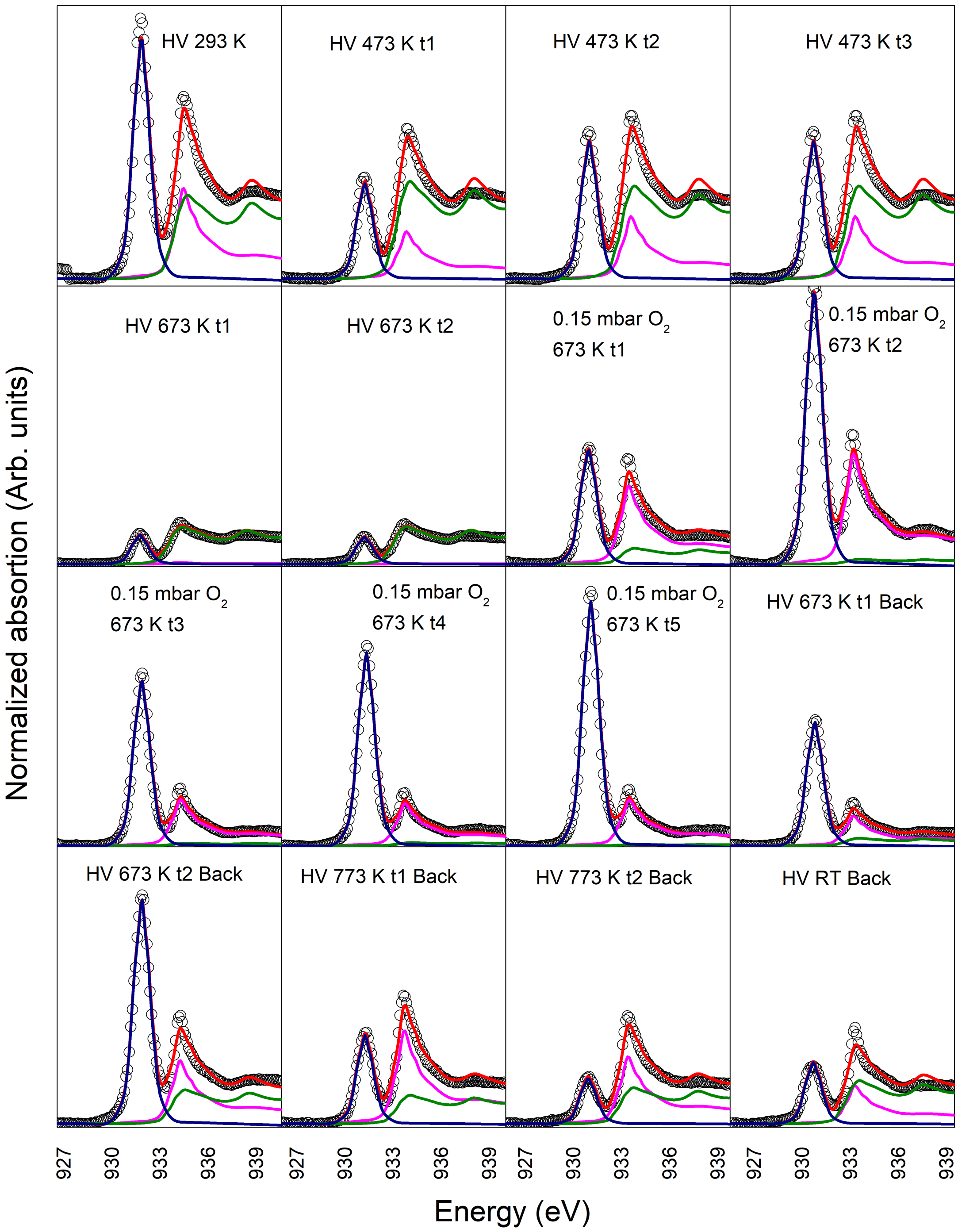

Supplementary Figure 8: **Linear combination fits of the Cu L_3-edge XANES spectra for all analyzed thermodynamic conditions for a sample with less than one monolayer of Cu_5 coverage.** Experimental data (open dots), linear combination fit (red line). CuO, Cu_2O and metallic Cu contributions are shown in blue, magenta and green lines, respectively. Each spectrum that is presented corresponds to a condition that was reached after waiting for the spectrum to not change.

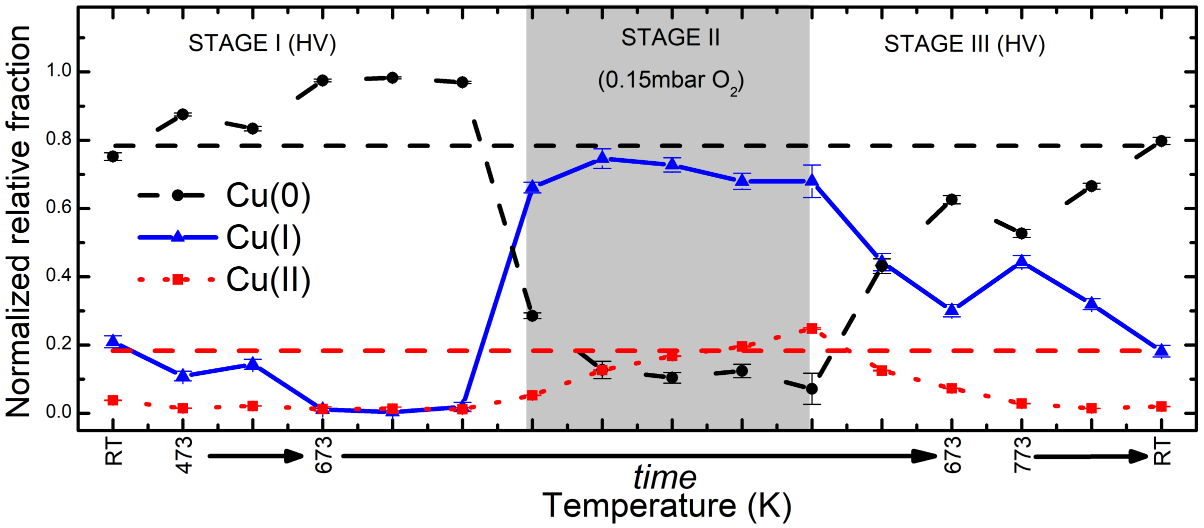

Supplementary Figure 9: **Relative fraction of Cu oxidation states of Cu_5/HOPG for a sample with less than one monolayer coverage in HV and 0.15 mbar of O_2 through the different stages of a cycle carried out from RT changing temperature and oxygen pressure**. Horizontal dotted lines indicate the initial concentration of the Cu(0) and Cu(I) states (shown with black and red colors, respectively). Error bars indicate the uncertainty of the linear combination fit algorithms used for the fitting procedure. Each spectrum used to obtain this figure corresponds to a condition that was reached after waiting for the spectrum to not change.

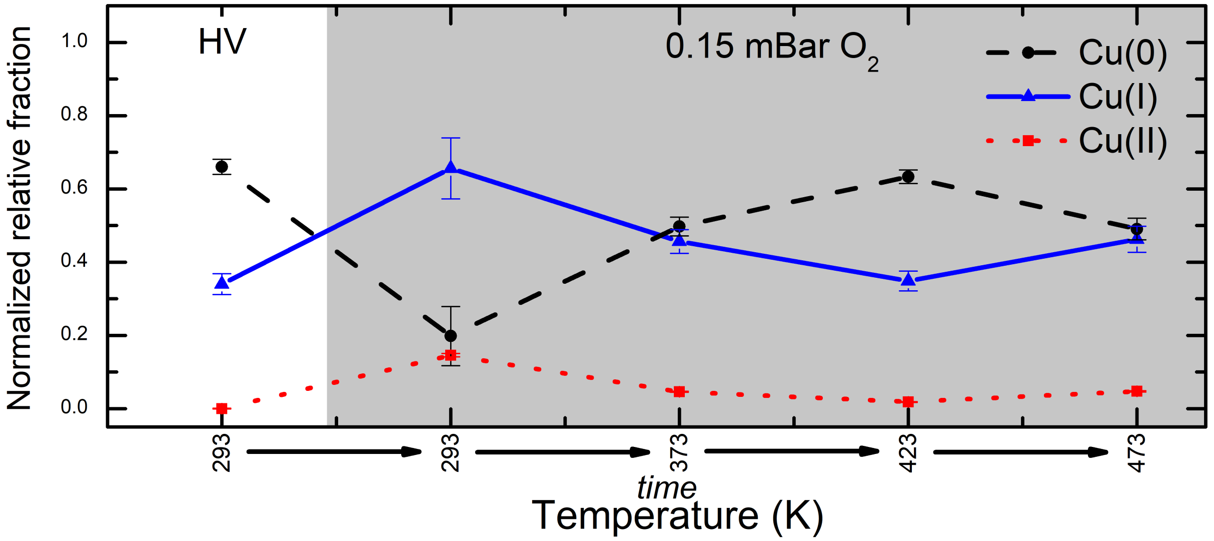

Supplementary Figure 10: **Relative fractions of Cu oxidation states upon oxidation/reduction cycling at low temperatures and oxygen pressure for a sample with less than one monolayer of Cu_5 coverage.**. The initial state of the system corresponds to the supported Cu_5 clusters on HOPG in high vacuum (white region) after a degassing treatment to remove the hydration shell from the mother's solution. Error bars indicate the uncertainty of the linear combination fit algorithms used for the fitting procedure. Each spectrum used to obtain this figure corresponds to a condition that was reached after waiting for the spectrum to not change.

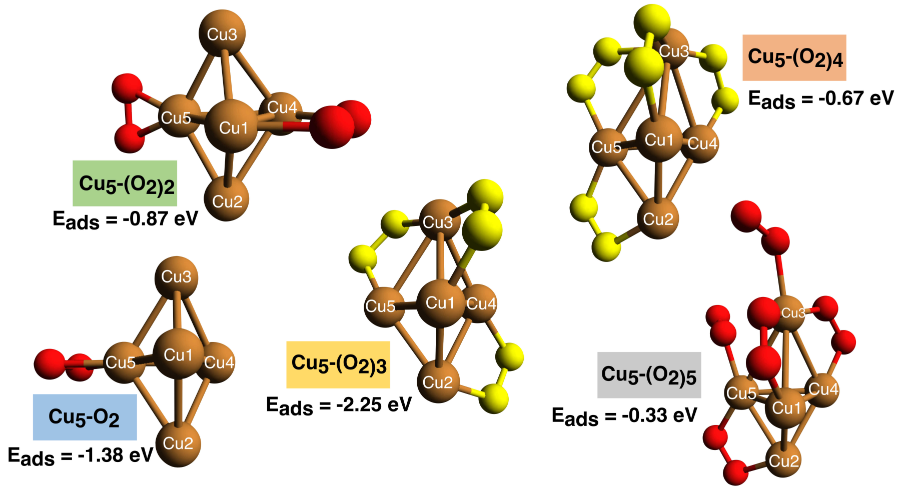

Oxidation state	Cu_5-O_2		Cu_5-$(O_2)_2$		Cu_5-$(O_2)_3$			Cu_5-$(O_2)_4$			Cu_5-$(O_2)_5$	
Level	DFT	HF	DFT	HF	DFT	HF	caspt2	DFT	HF	caspt2	DFT	HF
Cu1	0	0	+1	+1	+1	+1	+1/2	+3/4	+4/5	+4/5	+1	+1
Cu2	0	0	0	0	+1	+1	+1/2	+3/4	+4/5	+4/5	+1	+1
Cu3	0	0	0	0	+1	+1	+1	+2	+4/5	+4/5	+1	+1
Cu4	0	0	+1	+1	+1	+1	+1/2	+3/4	+4/5	+4/5	+1	+1
Cu5	+1	+1	+1	+1	+1	+1	+1/2	+3/4	+4/5	+4/5	+1	+1

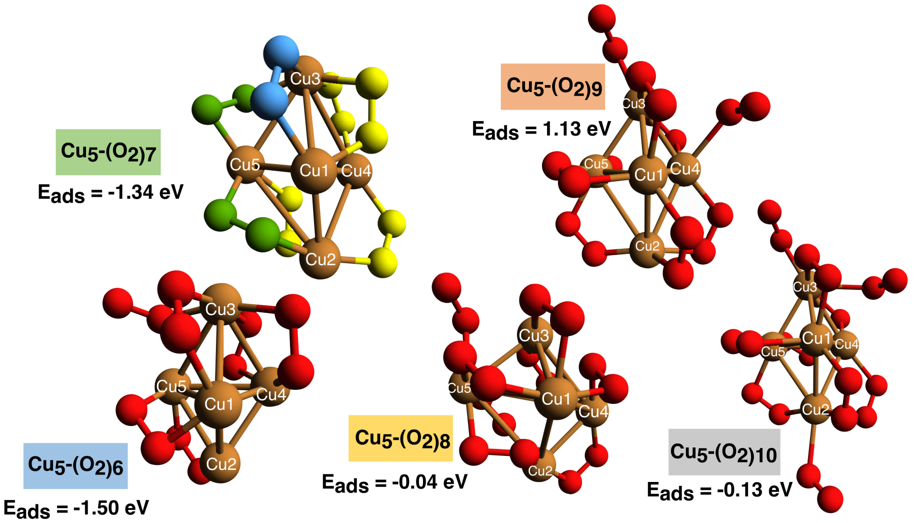

Oxidation state	Cu_5-$(O_2)_6$		Cu_5-$(O_2)_7$			Cu_5-$(O_2)_8$		Cu_5-$(O_2)_9$		Cu_5-$(O_2)_{10}$	
Level	DFT	HF	DFT	HF	caspt2	DFT	HF	DFT	HF	DFT	HF
Cu1	+2	+1	+1	+1	+1	+1	+2	+1	+1	+1	+1
Cu2	+1	+1	+2	+2	+2	+2	+2	+2	+1	+2	+1
Cu3	+2	+2	+2	+2	+2	+1	+1	+2	+1	+2	+1
Cu4	+2	+1	+2	+1	+1	+2	+2	+1	+1	+1	+1
Cu5	+2	+2	+2	+2	+2	+1	+2	+1	+1	+1	+1

Supplementary Figure 11: **Optimized structures, adsorption energies, and oxidation states of the copper atoms in Cu_5–$(O_2)_n$ complexes** ($n \leq 10$). Optimized stuctures and adsorption energies of Cu_5–$(O_2)_n$ complexes ($n \leq 10$), as calculated with the PBE-D3(BJ) approach and the def2-TZVP basis set. Assigned chemical oxidation states of copper atoms are also shown. The oxygen atoms colored in yellow, green, and blue highlight superoxo (O_2^-), peroxo (O_2^{2-}), and neutral O_2 species for the most stable complexes from the phase diagram (see Supplementary Section 10).

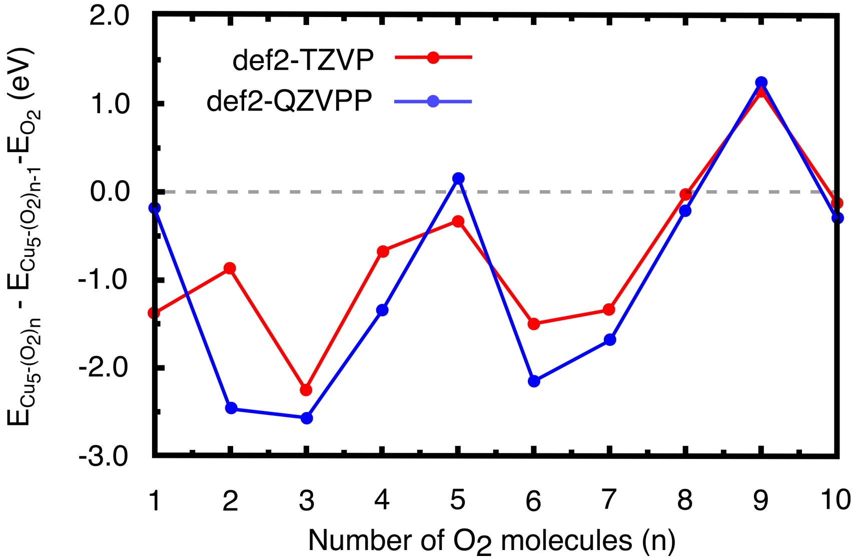

Supplementary Figure 12: **Adsorption energies as a function of the cluster size (n) for $(O_2)_n$–Cu_5 complexes.** Adsorption energies as a function of the cluster size (n) for $(O_2)_n$–Cu_5 complexes ($n \leq 10$), calculated at the PBE-D3(BJ) level of theory with the def2-TZVP (red) and the larger def2-QZVPP (blue) basis set.

a

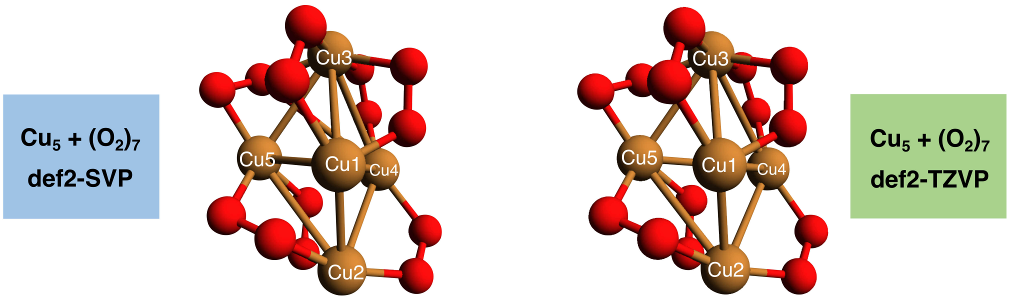

Distance (Å)	$Cu_5 + (O_2)_7$ def2-SVP	$Cu_5 + (O_2)_7$ def2-TZVP
**Cu1-Cu2**	2.543	2.535
**Cu1-Cu3**	3.081	3.059
**Cu1-Cu4**	2.852	3.014
**Cu1-Cu5**	2.751	2.790
**Cu2-Cu4**	2.939	3.007
**Cu2-Cu5**	3.356	3.361
**Cu3-Cu4**	3.173	3.227
**Cu3-Cu5**	3.022	2.981
**Cu4-Cu5**	3.051	3.242

b

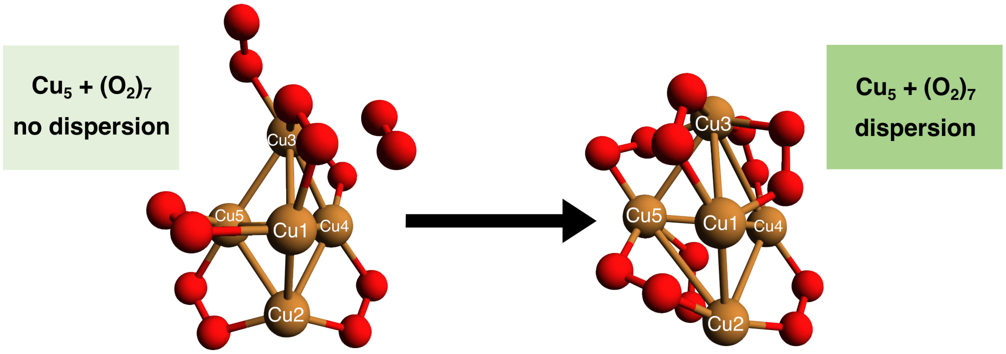

Distance (Å)	$Cu_5 + (O_2)_7$ no dispersion	$Cu_5 + (O_2)_7$ dispersion
**Cu1-Cu2**	2.552	2.535
**Cu1-Cu3**	2.827	3.059
**Cu1-Cu4**	2.566	3.014
**Cu1-Cu5**	2.483	2.790
**Cu2-Cu4**	2.512	3.007
**Cu2-Cu5**	2.594	3.361
**Cu3-Cu4**	2.573	3.227
**Cu3-Cu5**	2.686	2.981
**Cu4-Cu5**	2.612	3.242

Cu-Cu bond elongation

Supplementary Figure 13: **Convergence of structural parameters with the basis set size and effect of the dispersion interaction. a**, Comparison of the values of the Cu−Cu distances from the Cu_5–$(O_2)_7$ complex when calculated with the def2-SVP and def2-TZVP basis sets. **b**, Comparison of the values of the Cu−Cu distances from the Cu_5–$(O_2)_7$ complex when calculated without dispersion (left-hand structure) and adding the dispersion interaction (right-hand structure).

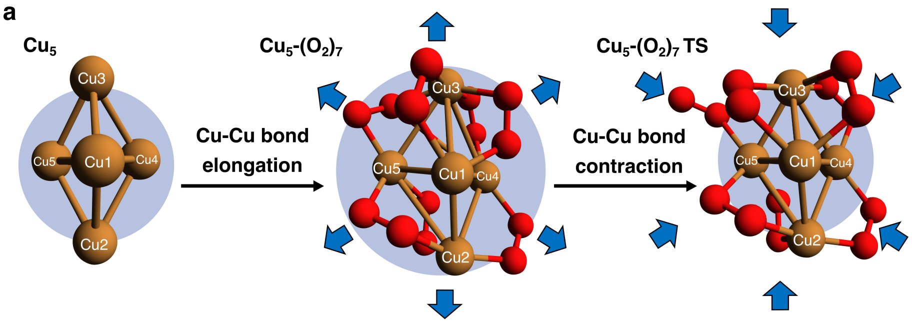

Distance (Å)	Cu_5	Cu_5-$(O_2)_7$	Cu_5-$(O_2)_7$ TS
**Cu1-Cu2**	2.455	2.535	2.491
**Cu1-Cu3**	2.458	3.059	2.731
**Cu1-Cu4**	2.352	3.014	2.560
**Cu1-Cu5**	2.564	2.790	2.660
**Cu2-Cu4**	2.440	3.007	2.577
**Cu2-Cu5**	2.456	3.361	2.934
**Cu3-Cu4**	2.439	3.227	2.682
**Cu3-Cu5**	2.456	2.981	2.550
**Cu4-Cu5**	2.361	3.242	2.604

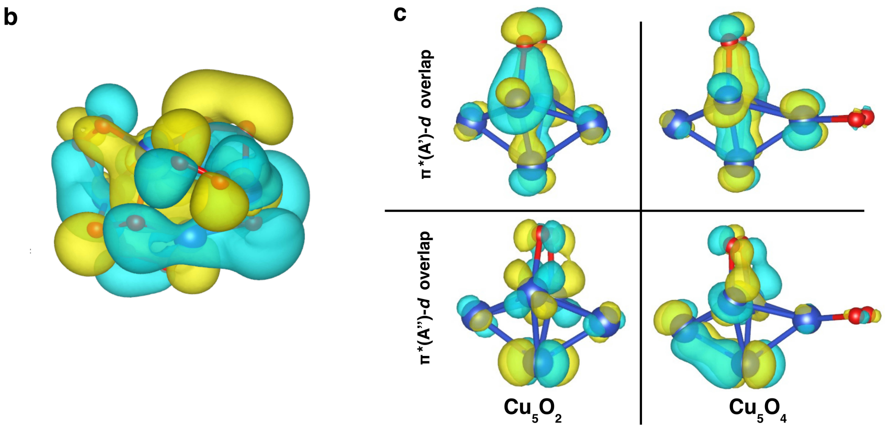

Supplementary Figure 14: **Collective effects in the adsorption of O_2 molecules to the Cu_5 cluster.** **a**, Figure showing how a Cu_5 cluster adsorbs and loses O_2 molecules by concerted elongations and contractions of Cu−Cu bonds. **b**, Picture of one occupied molecular orbital of the Cu_5–$(O_2)_7$ complex: the charge is collectively shared by the π^* orbitals of several O_2 molecules and collectively donated from $3d$ orbitals of most copper atoms. The pictured molecular orbital has significant projection on the $3d$ orbital of just one copper atom. **c**, Representative orbitals illustrating the nature of the $\pi^* - d$ bonding at the equatorial chemisorption (bridge) site in Cu_5–O_2 and Cu_5–$(O_2)_2$ complexes: the distortion of the $d-$orbitals arising from the absorption of a second oxygen molecule at the vortex site allows both $\pi^*(A')$ and $\pi^*(A'')$ orbitals of the equatorial O_2 molecule (the symmetric and anti-symmetric orbitals with respect to the equatorial plane of the cluster, respectively), to contribute to the bonding in the Cu_5–$(O_2)_2$ system. In contrast, the $\pi^*(A'')$ orbitals encompass negligible overlap with the $d-$orbitals from copper atoms in the Cu_5–O_2 complex. These orbitals have been obtained from *ab initio* calculations using multi-reference theory as described in the methods section.

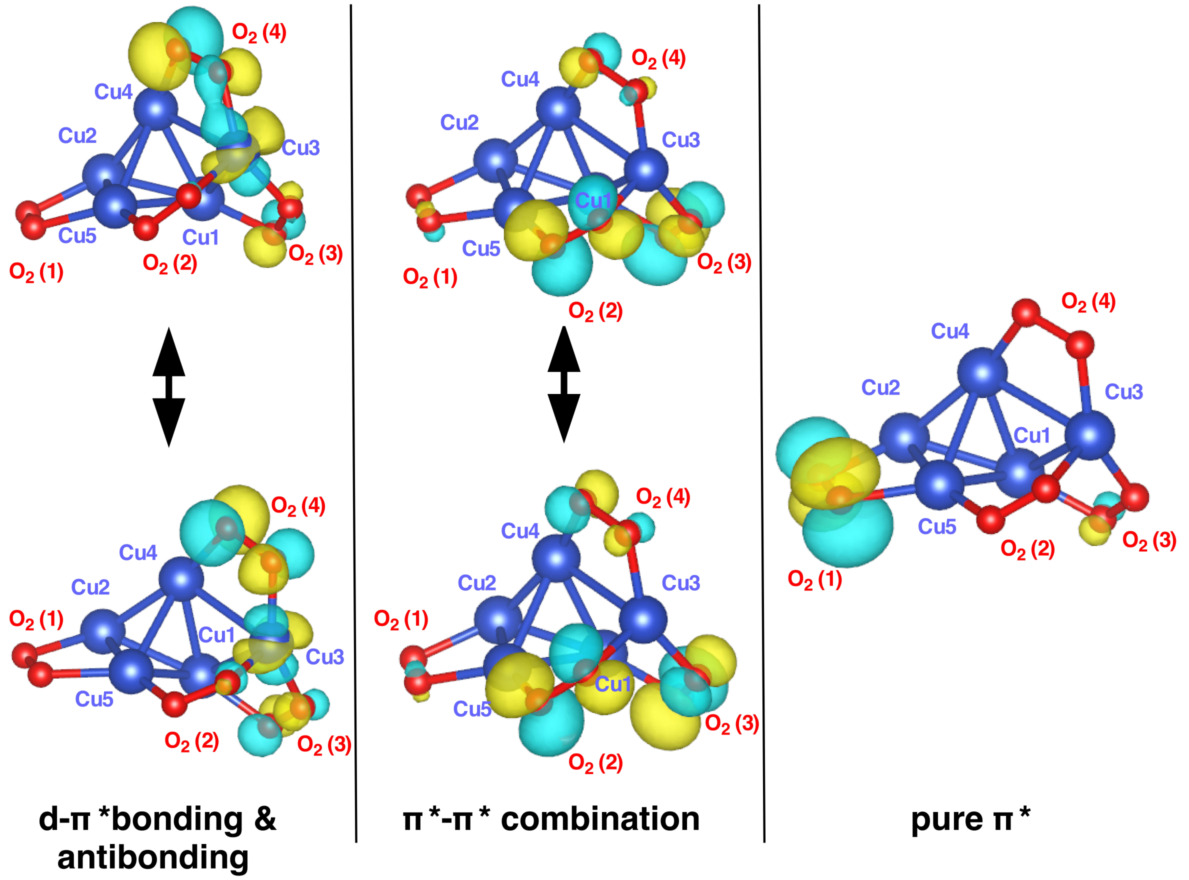

Supplementary Figure 15: **Characterization of the unpaired electrons of the Cu_5–$(O_2)_4$ complex.** Figure showing the five single-occupied orbitals of the Cu_5–$(O_2)_4$ system.

a

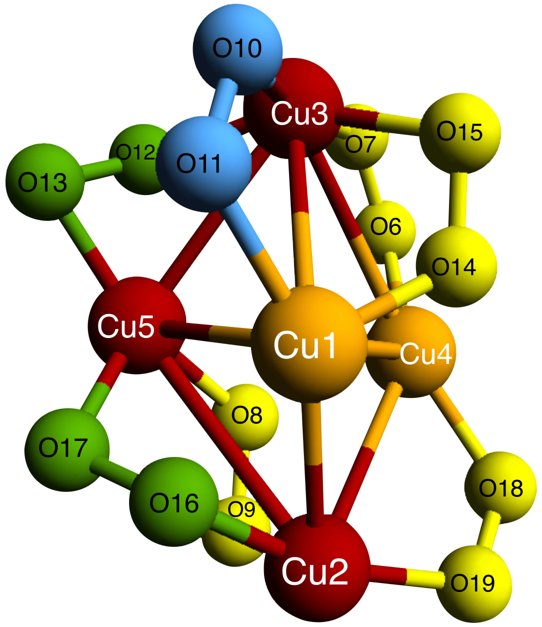

b

Cu atom	*s*	*p*	*d*	Oxidation state
**Cu1**	2.26	6.20	9.88	Cu(I)
**Cu2**	2.37	6.33	9.13	Cu(II)
**Cu3**	2.37	6.32	9.17	Cu(II)
**Cu4**	2.23	6.21	9.93	Cu(I)
**Cu5**	2.41	6.39	9.12	Cu(II)

c

O atom	*s*	*p*	*d*	Oxidation state
**O6**	3.88	4.25	0.05	superoxo
**O7**	3.87	4.50	0.04	
**O8**	3.89	4.59	0.04	superoxo
**O9**	3.91	4.15	0.04	
**O10**	3.90	4.10	0.04	neutral
**O11**	3.92	3.98	0.04	
**O12**	3.89	4.64	0.04	peroxo
**O13**	3.91	4.58	0.04	
**O14**	3.90	4.57	0.04	superoxo
**O15**	3.91	4.13	0.04	
**O16**	3.86	4.65	0.04	peroxo
**O17**	3.91	4.56	0.04	
**O18**	3.90	4.18	0.04	superoxo
**O19**	3.87	4.56	0.04	

Supplementary Figure 16: **Characterization of the oxidation states of Cu atoms from the Cu_5–$(O_2)_7$ complex**. **a**, Figure showing the structure of the Cu_5–$(O_2)_7$ complex and labelling of the different copper and oxygen atoms. Superoxo (O_2^-), peroxo (O_2^{2-}), and neutral O_2 molecules are represented with yellow, green, and blue balls, respectively. Copper atoms bearing Cu(II) and Cu(I) oxidation states are differentiated with red and brown colors. **b**, Mulliken population on atomic orbitals of copper atoms from the density matrix calculated for the Cu_5–$(O_2)_7$ complex using the CASPT2 method. The copper atoms are labelled as in Supplementary Figure 16a. **c**, Mulliken population on atomic orbitals of oxygen atoms from the density matrix calculated for the Cu_5–$(O_2)_7$ complex using the CASSCF method. The oxygen atoms are labelled as in Supplementary Figure 16a.

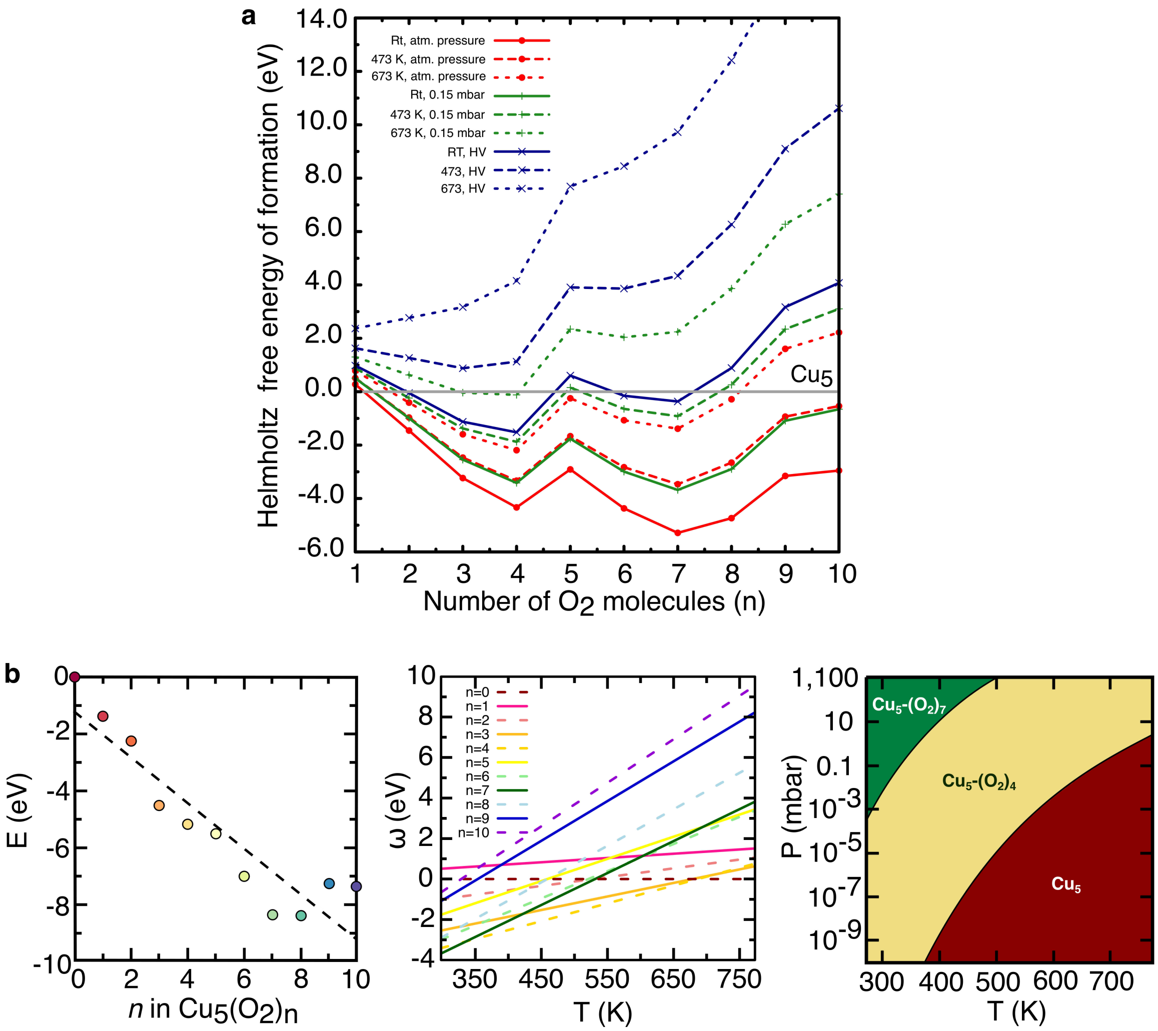

Supplementary Figure 17: **Helmholtz free energies of formation and phase diagram of Cu_5–$(O_2)_n$ complexes.** **a**, Free energies of formation of Cu_5–$(O_2)_n$ complexes, with the electronic energies and frequencies calculated using the def2-TZVP and def2-QZVPP basis sets, respectively, and the PBE-D3(BJ) approach. **b**, Left-hand panel: Electronic binding energies of the clusters as a function of the number of O_2 molecules. Middle panel: Free energy of formation (*w* potential) comparison at 0.15 mbar, with the color coding following dot colors in the left-hand panel. Even numbers of O_2 atoms are plotted as dashed lines. The predicted phases can be retrieved from the convex hull of the lowest energy curves. Right-hand panel: Final phase diagram, showing the most likely adsorption state as a function of pressure and temperature.

Supplementary Section 2: Tables

Supplementary Table 1: **Values obtained for the area under the Cu 2$p_{3/2}$ and C 1s peaks, area ratio, atomic ratio, and thickness. These values have been calculated as described by Hill et al.**[1]

	Monolayer	Concentrated
Cu 2$p_{3/2}$ Area (Arb. units)	677(3)	7589(9)
C 1s Area (Arb. units)	13000(10)	6675(8)
Cu/C Area ratio	0.052(5)	1.14(2)
Cu/C atomic ratio	0.16(2)	3.5(3)
Thickness (nm)	0.4(1)	4.3(2)

Supplementary Table 2: **Boltzmann-weighted averages of oxidation states (normalized fractions) at different values of temperature and oxygen pressure**. The averages have been calculated by applying the PBE-D3(BJ) approach with the def2-TZVP basis set for frequencies and the def2-QZVPP basis for electronic energies. The binding energies have been corrected for the basis set superposition error. Values between brackets have been obtained using the oxidation states estimated at *ab initio* (Hartree-Fock) level instead. At High-Vacuum (HV), the probability of trapping at the physisorption state has also been considered[2] (values between parenthesis).

Pressure	T	Cu(0)	Cu(I)	Cu(II)
1 atm	RT	0.0	0.20	0.80
		[0.0]	[0.40]	[0.60]
1 atm	473 K	0.01	0.22	0.77
		[0.01]	[0.42]	[0.57]
0.15 mbar	RT	0.0	0.20	0.80
		[0.0]	[0.40]	[0.60]
0.15 mbar	373 K	0.20	0.60	0.20
		[0.20]	[0.80]	[0.0]
0.15 mbar	423 K	0.20	0.60	0.20
		[0.20]	[0.80]	[0.0]
0.15 mbar	473 K	0.20	0.60	0.20
		[0.20]	[0.80]	[0.0]
0.15 mbar	673 K	0.24	0.61	0.15
		[0.31]	[0.69]	[0.0]
0.15 mbar	773 K	1.0	0.0	0.0
		[1.0]	[0.0]	[0.0]
HV	RT	0.20	0.60	0.20
		(0.87)	(0.10)	(0.03)
		[0.20]	[0.80]	[0.0]
		(0.87)	(0.13)	(0.0)
HV	473 K	1.0	0.0	0.0
		[1.0]	[0.0]	[0.0]
HV	673 K	1.0	0.0	0.0
		[1.0]	[0.0]	[0.0]
HV	773 K	1.0	0.0	0.0
		[1.0]	[0.0]	[0.0]

Supplementary Section 3: XPS quantification of Cu_5 clusters deposited on HOPG

Aimed to compare different final concentrations of Cu_5 clusters deposited on HOPG, we estimated the Cu to C atomic ratio by using the XPS survey spectra of the samples under study (see Supplementary Figure 3). Due to the possible carbon contamination, the resulting estimate of the Cu to C atomic ratio could be undervalued. Yet, the carbon contamination signal could be safely discarded when it was compared to that of pure carbon in HOPG. The intensity of the Cu $2p_{3/2}$ and C $1s$ levels in these spectra was determined by integration once the background was properly subtracted (see Supplementary Table 1).

Just by comparing the ratio between the Cu $2p_{3/2}$ and C $1s$ intensities for each sample, it is possible to determine that the sample referred to as "concentrated" (used in the Cu K-edge XANES and NAP-XPS experiments) has a Cu_5 clusters concentration $\sim$ 20 times larger than the so-named "monolayer" sample. These intensities were corrected by the proper relative sensitivity factor (RSF) in order to take into account the electron mean free path, cross section, and other experimental factors which are usually included to precisely estimate the atomic concentration by XPS.[3] These corrections allows to determine the copper to carbon atomic ratio summarized in Supplementary Table 1.

Using the intensities corrected by the RSF factors, it is possible to estimate the thickness of the Cu overlayer (see Supplementary Table 1).[1] This estimation, besides its simplicity, provides us with a raw estimation of the number of layers in each sample, being close to 1 for the "monolayer" sample and about 10 for the "concentrated" one. To get these values, it was assumed that the Cu_5 clusters size is that estimated by HAADF-STEM (approximately 0.4 nm), also described in the theoretical section.

Supplementary Section 4: Cu *K*-edge XANES experiments in air (high concentration of Cu_5 clusters)

Supplementary Figure 4 shows the complete set of Cu *K*-edge XANES spectra of Cu_5 clusters deposited on HOPG, measured at air and obtained with the following sequence: heating the sample from room temperature (RT) up to 473 K, cooling down to RT and then heating up to 673 K. All spectra exhibit the characteristic features of Cu atoms in the Cu(II) state, with the energy edge position at 8986 eV and a weak feature (pre-peak) located at 8979 eV. Supplementary Figure 5 shows the XANES spectra during the first heating process between RT and 473 K. Some changes are observed during this first stage. The most important one takes place at around 348 K, a temperature at which the edge position is slightly shifted by 1 eV towards lower energies. This shift is usually a consequence of the reduction of the Cu average oxidation state. However, Zhang et al.[4] have shown that a decrease in the coordination number under absorption of H_2O on Cu(II) causes a small shift to lower energies of the edge and changes in the feature at 8986 eV. Thus, the modifications observed in the XANES spectra during heating from RT to 348 K must be attributed not to a change in the oxidation state but to the desorption of the hydration shell from mother solution and/or oxygen (degassing) during this first treatment.

XANES measurements at the Cu *K*-edge in air have shown that when the concentration of Cu_5 clusters on the HOPG surface is high (about ten monolayers according to the XPS experimental estimations, see Supplementary Figure 3), their Cu_5 clusters identity is lost due to the formation of bulk CuO when the temperature is increased above 673 K.

Supplementary Section 5: Near Ambient Pressure XPS experiments at different oxygen pressures

Cu 2*p* XPS spectra were taken at different pressures of oxygen at the Near Ambient Pressure XPS (NAP-XPS) main chamber. The purpose of these measurements was to identify the best choice of pressure for detecting oxidized Cu species without a significant signal attenuation due

to the presence of the gas phase. Supplementary Figure 6 shows the corresponding Cu 2*p* XPS spectra for oxygen pressures in the range from 1.5×10^{-6} mbar to 1.5 mbar. Clearly, a pressure of the order of 0.15 mbar is high enough to form and detect the presence of oxidized Cu(II) species.

Supplementary Section 6: Cu L_3-edge XANES experiments in high vacuum and in 0.15 mbar of oxygen (low concentration of Cu_5 clusters)

Cu L_3-edge XANES measurements were performed on monolayer-like samples of Cu_5 clusters on HOPG. For these experiments, the temperature was varied between 293 K (RT) and 773 K, in high-vacuum (HV) and in 0.15 mbar of oxygen pressure. In order to identify and quantify the different oxidation states and their relative concentration in the sample at different thermodynamic conditions, a linear combination fit of these XANES spectra was performed, following the procedure described by Eren et al.[5] Spectra of metallic Cu, Cu_2O and CuO were used as reference for Cu(0), Cu(I) and Cu(II) oxidation states, using the reported values of the Cu L_3-edge energies of 933.7, 933.7, and 931.3 eV, respectively.[6] In fact,it is already accepted that studies of the Cu L_3 absorption edge offers an excellent method to determine the presence of mono and divalent copper in solids. Divalent compounds have an incompletely filled *d* band, and almost all the intensity is found in a single line around 931 eV. The method corresponds to an empirical fingerprint approach, justified on experimental evidence, which is commonly accepted and employed for similar purposes. Examples are Ref. 7, where the authors determine the Cu oxidation state in catalytic reactions by using Cu-oxide reference compounds employing in situ Cu L-XANES spectroscopy, or Ref. 8 where the authors use the Cu $2p \rightarrow 3d$ L-edge intensity to experimentally obtain the amount of unoccupied metal *d* character in the ground state wave function and thus quantify the amount of Cu(II) in $[CuCl_4]^{2-}$ complexes. The intensity of the Cu L_3 absorption edge is strongly influenced by the chemical state of Cu atoms.[6] Supplementary Figure 7 shows the Cu L_3-edge XANES spectra for the three reference compounds together with that of the Cu_5 clusters on HOPG sample in HV after heating in vacuum at 673 K. CuO and Cu_2O have strong

absorption edges at 931.3 and 933.7 eV, respectively, and substantial shape differences between them and the corresponding spectrum of metallic Cu.

All the Cu L_3-edge XANES spectra collected at the different thermodynamic conditions and their corresponding fits are presented in Supplementary Figure 8. Finally, Supplementary Figure 9 shows the relative fractions of oxidation states of Cu atoms in Cu_5 clusters through the different stages of a cycle carried out from RT to 773 K at HV and 0.15 mbar of oxygen pressure (see Figure 3).

Aimed to reexamine and quantify the observed interaction between (molecular) oxygen and Cu_5 clusters by NAP-XPS, we performed Cu L_3-edge XANES experiments, fixing the oxygen pressure at 0.15 mbar, between RT and 473 K. This way, we confirmed that the Cu_5 clusters are reduced, even in the presence of (0.15 mbar) of oxygen. As aforementioned (see Figure 2c), the oxidation state reduction of Cu_5 clusters proceeds at moderated temperatures (in the range from 373 to 473 K) in presence of oxygen. The analysis of these measurements, shown in Supplementary Figure 10, not only corroborates the previous findings by NAP-XPS but also allows a "quantification" of the reduction mechanism. This way, the determined fractions of Cu oxidation states can be directly compared with the theoretical predictions in a wider range of thermodynamic parameters (see Figure 5). The key result is a clear confirmation of the reduction of the oxidation state of the Cu_5 clusters in presence of 0.15 mbar of oxygen, after having been oxidized at RT under the same atmosphere.

Supplementary Section 7: Methods used in the theory part

In all calculations on bare Cu_5 clusters, a trigonal bipyramidal (3D) structure is assumed. Although the experimental measurements have been realized for Cu_5 clusters supported on highly oriented pyrolytic graphite (HOPG), a previous work[9] has shown that a Cu_5 cluster is only minimally perturbed when supported on a carbon-based surface (graphene) due to the dispersion-dominated nature of the Cu_5–graphene interaction. Specifically, using a dispersion-corrected DFT approach[10,11] with the computational setup reported in Refs. 12,13 to describe the Cu_5/graphene interaction,[9] it was found from a Bader decomposition analysis[14] that the net charge donation

from the copper cluster to the support is insignificant (less than 0.02 $|e|$). Therefore, the experimental results for HOPG-supported Cu_5 clusters are considered as representative of unsupported Cu_5 clusters.

Density functional theory (DFT) as well as multi-reference perturbation theory[15] have been applied to study the chemisorption of up to 10 O_2 molecules on Cu_5 clusters. To this end, we have employed a dispersion-corrected DFT-D3 ansatz,[10,11] given its well-proven performance in describing the adsorption of small silver clusters on a titanium dioxide (TiO_2) surface[16,17] and the optical properties of the same surfaces when modified via deposition of Cu_5 clusters.[9] Specifically, structural optimizations and the calculation of interaction energies have been performed with the Perdew-Burke-Ernzerhof (PBE) density functional and the Becke-Johnson (BJ) damping[10] for the D3 dispersion correction. This combination will be referred to as the PBE-D3(BJ) scheme. These calculations have been performed with the ORCA[18] suite of programs (version 4.0.1.2). The optimization of the geometries of the Cu_5– $(O_2)_n$ complexes have been accomplished using the atom-centered def2-TZVP[19] basis set for copper and oxygen atoms. Additional single-point calculations were carried out using the def2-QZVPP basis set.[19] The counterpoise method was employed to estimate the basis set superposition error.[20] Single-point calculations have been also performed at Møller-Plesset perturbation theory (MP2) level with the def2-SVP[19] basis set, and at Hartree-Fock (HF) level using the def2-TZVP[19] basis set. All the DFT calculations were carried out considering the doublet spin states of the Cu_5–$(O_2)_n$ complexes.

We have also applied the single-state CASSCF method[15] with density fitting (DF-CASSCF), as implemented within the MOLPRO program package.[21] For this purpose, we used the polarized correlation-consistent triple-ζ basis of Dunning and collaborators[22] (cc-pVTZ) for oxygen atoms, and the cc-pVTZ-PP basis set for copper atoms[23] including a small (10-valence-electron) relativistic pseudopotential. For density fitting, the associated JKFIT bases was applied.

Supplementary Section 8: Molecular chemisorption states of Cu_5–$(O_2)_n$ complexes

In order to consider a large number of O_2 molecules adsorbed on the Cu_5 cluster, we have applied density functional theory as described in the Methods section. The optimized geometries of Cu_5–$(O_2)_n$ complexes are shown in Supplementary Figure 11, as calculated with the PBE-D3(BJ) approach and the def2-TZVP basis set. The values of the adsorption energies as a function of the number of O_2 molecules are indicated in Supplementary Figure 11 (see also Supplementary Figure 12). The optimization of geometries has been performed using both the def2-SVP and the def2-TZVP basis sets in order to ensure convergence in the values of the structural parameters (see Supplementary Figure 13a). Since minor modifications are found in structural parameters such as Cu−Cu distances when using def2-SVP and def2-TZVP basis sets (see Supplementary Figure 13a), the resulting optimized structures are expected to be rather well converged when using the def2-TZVP basis. Therefore, this basis was used to calculate the frequencies necessary for thermochemistry (see Section 10).

Binding energies E_b are defined as

$$E_b = E_{\mathrm{Cu_5-(O_2)}_n} - E_{\mathrm{Cu_5}} - n \cdot E_{\mathrm{O_2}} \tag{S1}$$

and have been calculated using the def2-TZVP and def2-QZVPP basis sets. Upon augmenting the basis set, binding energies become ca. 0.18 eV lower in average with the largest (def2-QZVPP) basis set, while the inclusion of the Boys-Bernardi counterpoise correction[20] made them ca. 0.22 eV higher. Hence, the values of the binding energies are considered to be also well converged using the latter. The same holds true with the values of the adsorption energies E_{ads} as shown in Supplementary Figure 12, which are defined as

$$E_{\mathrm{ads}} = E_{\mathrm{Cu_5-(O_2)}_n} - E_{\mathrm{Cu_5-(O_2)}_{n-1}} - E_{\mathrm{O_2}} \tag{S2}$$

The analysis of Supplementary Figure 11 (see also Supplementary Figure 12) points out the

enhanced stability of the Cu_5–$(O_2)_n$ complexes for which the O_2 molecules locate at bridge positions of the Cu_5 cluster. Less stable structures involve the attachment of O_2 molecules to one copper atom only. For example, as can be observed in Supplementary Figure 11, the very small adsorption energy (less than -0.05 eV) of one additional O_2 molecule to the Cu_5–$(O_2)_7$ complex indicates that it becomes physisorbed. The addition of further O_2 molecules involves either positive or very small adsorption energies (ca. 0.1 eV as much), indicating the lack of stability of complexes with $n > 7$.

Notice from Supplementary Figure 13a that the Cu−Cu bond lengths increase in comparison to those of the bare Cu_5 cluster upon adsorption (see also Supplementary Figure 14). According to the atomic spin populations, the O_2 molecules become either superoxo or peroxo species, which is also reflected in the enlargement of the O−O bond lengths (larger than ca. 1.4 Å) upon adsorption from the value for the neutral O_2 molecule (ca. $\sim$ 1.2 Å). As shown in Supplementary Figure 13b, it is necessary to add the dispersion interaction in getting the right structure for the complex bearing the maximum number of O_2 molecules adsorbed on bridge sites (i.e, the Cu_5–$(O_2)_7$ complex). The inclusion of the dispersion is in fact correlated with the enlargement of the Cu−Cu bonds, as necessary to accommodate peroxo and superoxo species with larger O−O bond lengths than for the neutral O_2 molecule.

We also compared adsorption energies obtained at DFT and MP2 levels of theory, finding that the complexes with 3 and 7 O_2 molecules are the most stable at 0 K. For the complex with 7 O_2 molecules, the MP2 adsorption energy was ca. 0.5 eV lower than the DFT value using the def2-SVP basis set. Using the def2-TZVP basis set instead, the energy difference between MP2 and DFT values was also found to be ca. 0.5 eV for the Cu_5–O_2 complex. Considering that the single-point MP2 calculations are performed on top of optimized structures via DFT and not MP2 calculations, energy differences of ca. 0.5 eV are sensible, indicating that the DFT approach is sufficient in getting reasonable values of adsorption and binding energies.

The Cu_5 cluster can be thought as a network in which all atomic nuclei and electrons motions are collectively correlated. For instance, as can be seen in Supplementary Figure 14a, the Cu_5 cluster adsorbs and loses O_2 molecules by concerted elongations and contractions of the Cu−Cu bonds. The same holds true for the motion of electrons: as illustrated in Supplementary Fig-

ure 14b, the electronic charge is collectively shared by all O_2 molecules and mainly located in their π^* orbitals. It is interesting that this collective effect favors the charge transfer from the Cu_5 cluster to the O_2 molecules when the number of O_2 molecules increases. For instance, when only one O_2 is chemisorbed at the bridge adsorption site located at the equatorial plane of the Cu_5 cluster, only a partial charge transfer is observed.[24] On one hand, this partial charge transfer arises from the very favorable overlap of the π^* orbital from the oxygen atoms, located at the equatorial plane of the cluster, with several $d-$type orbitals of the copper atoms. This special orbital from the adsorbed O_2 molecule will be referred to as the $\pi^*(A')$ orbital since it bears A$'$ symmetry with respect to the equatorial plane of the cluster. As a representative example, the interaction of this $\pi^*(A')$ orbital with one of the d-type orbitals from the copper atoms is depicted in Supplementary Figure 14c. On the other hand, the $\pi^*(A'')$ orbital (which is anti-symmetric with respect to the equatorial plane) barely overlaps with the d-type orbitals and does not contribute to the bonding, as can be seen in the left-hand panel of Supplementary Figure 14c. Importantly, when a second O_2 molecule becomes attached to the Cu_5 cluster at its vortex adsorption site, the whole structure of the d-type orbitals from copper atoms becomes distorted. This distortion does not affect the favorable overlap of the $\pi^*(A')$ orbitals, but it allows a more favorable interaction of the $\pi^*(A'')$ orbitals with the d-type orbitals of the Cu_5 cluster, as shown in the right-hand panel of Supplementary Figure 14c. Since a similar effect occurs not only for the represented orbitals, but also for many of them, the bonding between both the π^* orbitals of the O_2 molecules adsorbed on the bridge sites with several d-type orbitals from the copper atoms allows the oxygen molecules to drain more efficiently electrons from the copper atoms leading to net charge transfers. This effect is then additive for each additional oxygen molecule which becomes adsorbed to the Cu_5 cluster. Notice that the collective effects in the atomic nuclei and electrons motions are possible due to the subnanometer-sized network formed by the $3d$-orbitals of the copper atoms.

Supplementary Section 9: Oxidation states of the copper atoms: multireference theory

To provide measurable predictions, we have assigned oxidation [Cu(0), Cu(I), Cu(II)] states to the copper atoms of each Cu_5–$(O_2)_n$ complex since experimental measurements have provided relative Cu oxidation states at different values of temperature and pressure. Assigned oxidation [Cu(0), Cu(I), and Cu(II)] states for each Cu_5–$(O_2)_n$ complex are indicated in Supplementary Figure 11, as deduced from an analysis of Mulliken charges[25] and atomic spin populations using the Hirshfeld method.[26,27] For the purpose of comparison, this analysis has been realized at both the PBE-D3(BJ) and the Hartree-Fock level of theory.

To provide a more accurate analysis of the the nature of the chemical oxidation states of copper atoms for the most stable Cu_5–$(O_2)_n$ complexes, we have also applied high level multireference *ab initio* theory. It should be stressed that the peroxo or superoxo character of the adsorbed O_2 molecules as well as differential effects in Cu(I) and Cu(II) oxidation states could be assigned even at the HF level. A Hirshfeld analysis of atomic spin populations revealed that a characteristic of the most stable Cu_5–$(O_2)_4$ and Cu_5–$(O_2)_7$ complexes is in bearing a net unpaired electron on the $3d$ orbitals of one ($n = 4$) or three ($n = 7$) copper atoms. This is one of the signatures of the possible occurrence of Cu(II) oxidation states since the electronic configuration of bare copper atoms (i.e., in the Cu(0) state) can be approximated to a first-order as: $[Ar](3d)^{10}(4s)^1$, with [Ar] denoting the electronic configuration of argon. However, the Hartree-Fock method is still a single-reference approach. To properly account for the open-shell nature of the interacting systems, multireference perturbation theory has been applied. These calculations were computationally very expensive, encompassing hundreds of millions of configuration state functions. Both doublet and quartet spin states were considered, finding them almost degenerate as expected: the total energy is kept almost unperturbed upon the spin-flip of unpaired electrons located on distant superoxo O_2^- radicals (from, e.g., anti-ferromagnetic to ferromagnetic configurations).

Supplementary Section 9.1 Cu_5–$(O_2)_4$

The correlated analysis of spin atomic populations (Hirshfeld analysis) and Mulliken charges of the natural orbitals reveals that the Cu_5–$(O_2)_4$ cluster oxide bears five unpaired electrons when four O_2 molecules are chemisorbed. To describe properly a doublet spin wave-function encompassing five unpaired electrons and estimate the correct charge distribution, it is mandatory to use multireference methods. For this system, the active space considered in the CASSCF calculation consists of 13 electrons in 9 orbitals and 73 orbitals being closed. From the analysis of the multi-reference wave-function, it is clear that all O_2 molecules in the Cu_5–$(O_2)_4$ complex can be characterized as superoxo O_2^- radicals, with a spin of almost unity for each of them and a Mulliken charge of ca. -0.7 $|e|$. It is known that the Mulliken analysis generally underestimates the ionicity degree of the binding, but remaining globally proportional to the real charge distribution. Since the four oxygen molecules present an unitary spin, it can be assumed that they strip four electrons (one each) to the Cu_5 cluster. The copper atom coordinated with three oxygen atoms (labelled as Cu3 in Supplementary Figure 15) is the atom having a single-occupied $3d$ orbital. Its electronic configuration can be roughly approximated as $[\mathrm{Ar}](3d)^9(4s)^0$. Sharing one electron from one $3d$ orbital makes its binding with three oxygen atoms possible. This electronic configuration for a single copper atom would suggest that it presents a Cu(II) oxidation state. However, since it bears the same value of the Mulliken charge as the four O_2 molecules, but of opposite sign, it is clear that its oxidation state is Cu(I). This is due to the ability of the cluster to redistribute the charge towards other copper atoms through a complex binding network formed by the $d-$orbitals. As a consequence of this positive charge delocalization, the other vortex copper (labelled Cu2 in Supplementary Figure 15) bears the same Mulliken charge and can be also assigned with a Cu(I) oxidation state, even if coordinated to just one oxygen atom. The two remaining positive charges are delocalized over the three equatorial copper atoms with an oxidation state which is intermediate between the Cu(0) and Cu(I) states (i.e., approximately +2/3).

The fact that the copper cluster becomes the carrier of four superoxo radicals is also reflected on the spin degeneracy of doublet and quartet spin states due to the small magnetic coupling

between unpaired electrons located in π^* orbitals of distant O_2 molecules. Thus, energy differences below 0.01 eV were found in the the CASPT2 calculations, involving about 250 millions of configuration state functions. The wavefunctions were found to be highly multi-configurational, encompassing ca. 22 configuration state functions with coefficients larger than 0.1 for the doublet spin state. Hence, an analysis of the natural orbitals from such multi-configurational wavefunction was mandatory to ensure the identification of the nature of copper oxidation states.

The five singly-occupied orbitals of the system are represented in Supplementary Figure 15. A simple molecular orbital model is capable of explaining the nature of the copper oxidation states. As can be clearly seen from Supplementary Figure 15, one electron is located on a pure π^* orbital of one O_2 molecule (shown at the right-hand panel), with its other π^* orbital being filled (i.e., double occupied). Hence, it is evident that one negative charge is localized on this molecule.

It can be also observed in Supplementary Figure 15 that two unpaired electrons are located in two orbitals which are essentially linear combination of two π^* orbitals from the second and third oxygen molecules, with a small contribution from the fourth (shown in the middle panel). In a first-order approximation, two unpaired electrons which are delocalized on two sites can be thought as one electron on each oxygen molecule, with their other π^* orbitals being filled for both O_2 molecules. Hence, it is clear that each O_2 molecule bears one negative charge as well.

Finally, the two remaining unpaired electrons are located in two molecular orbitals which are combinations between one d-orbital of the copper atom labelled as Cu_3 in Supplementary Figure 15 and one π^* orbital from the fourth oxygen molecule. One molecular orbital is bonding and the other one is anti-bonding, with both having a small contamination of the π^* orbital from the third oxygen molecule. In a first-order approximation, the two unpaired electrons are thus located on the fourth oxygen molecule and on the vortex Cu atom coordinated with three oxygen atoms, with the other π^* orbital of this fourth oxygen molecule being filled too. Thus, it is apparent that the fourth O_2 molecule bears one negative charge.

It is noticed that the bare Cu_5 cluster conserves an unpaired electron. However, instead of being delocalized in several s-type orbitals as for the isolated cluster, it becomes localized in one d-type orbital. This qualitative model based on interpretation of the molecular orbitals leads

to the conclusion that the Cu_5 cluster should carry four positive charges spread over five copper atoms, giving a mean value of 0.8 $|e|$. This is in agreement with the assignment from the Mulliken population analysis, which predicts two copper atoms having the Cu(I) oxidation state, with two positive charges distributed over three copper atoms located at the equatorial plane.

In order to emphasize the importance of an appropriate multi-reference treatment in characterizing properly the charge distribution of this system, we mention that our DFT calculations using the largest (def2-QZVPP) basis set provided a prediction of the net charge transfer of ca. 1.4 $|e|$ only and no net (almost unity) spin population on copper atoms whatever the complex size be.

Supplementary Section 9.2 Cu_5–$(O_2)_7$

The active space considered for this system consists in 11 electrons in 10 orbitals, with 98 closed orbitals. The analysis of the density matrix from the multi-configurational wave-function for the Cu_5–$(O_2)_7$ complex clearly reveals a Cu(II) oxidation state for three copper atoms (labelled as Cu_2, Cu_3, and Cu_5 in Supplementary Figure 16a) while the other two copper atoms can be characterized with the Cu(I) oxidation state (labelled as Cu_1 and Cu_4 in Supplementary Figure 16a). Obviously, copper atoms having higher oxidation states are those coordinated to more oxygen atoms. The adsorbed O_2 molecules can be clearly characterized as either superoxo or peroxo radicals, with one neutral O_2 molecule having been identified as well. As for the Cu_5–$(O_2)_4$ complex, the quartet and doublet spin states were found to be almost degenerate (to within 0.01 eV).

As can be observed in Supplementary Figure 16a, copper atoms in assigned Cu(II) oxidation states are characterized by having, approximately, one single-occupied 3d orbital and an unoccupied 4s orbital. Actually, the loss of electronic charge from 3d ($0.8-0.9$ $|e|$) and 4s (ca. 0.6 $|e|$) orbitals is not unity, when analyzed with the Mulliken analysis. The analysis of spin atomic populations is even more conclusive, predicting the copper atoms in Cu(II) oxidation states as having a spin population close to unity. Moreover, they bear about the same value of the Mulliken charge as the peroxo radicals but opposite sign. However, from Supplementary Figure 16b, an increase of the electronic charge located in p-type orbitals can be also observed ($0.2-0.4$ $|e|$),

which is attributed to a back-donation transfer process from the O_2 molecules to the Cu_5 cluster.

As shown in Supplementary Figure 16c, O_2 molecules adsorbed as superoxo (O_2^-) and peroxo (O_2^{2-}) radicals receive a Mulliken charge donation of ca. 0.6 and 1.1 $|e|$, respectively. This excess electronic charge is accommodated in atomic p-type orbitals. Our assignment is confirmed from the spin atomic population analysis using the Hirshfeld method. Notice that the electronic population of s-type orbitals decrease by ca. 0.1 $|e|$ per oxygen atom. Hence, a back-donation transfer of electronic charge is predicted from s-type orbitals of adsorbed O_2 species to p-type orbitals of the Cu_5 cluster (see Supplementary Figure 16b). The net charge donation from the Cu_5 cluster, as estimated through the Mulliken populations, is close to 5 $|e|$ in total. Once again, this value is smaller than expected from the formation of 4 superoxo and 2 peroxo radicals (i.e., 8 $|e|$). Besides the well-known underestimation of electronic charge transfer using the Mulliken analysis, there is also a clear back donation from the O_2 molecules to the Cu_5 cluster (ca. 1.4 $|e|$).

The multi-configuration nature of the wavefunction for the Cu_5–$(O_2)_7$ complex is also worth stressing, encompassing ca. 20 configurations with coefficients larger than 0.05. Remarkably, the Hartree-Fock method is already capable of providing correct values of the oxidation states for copper atoms via the Hirshfeld analysis of their spin atomic populations. Thus, the three copper atoms highlighted in red in Supplementary Figure 16a carry spins close to unity.

Supplementary Section 10: Helmholtz free energies of formation and phase diagram of Cu_5–$(O_2)_n$ complexes

At given temperature (T) and partial oxygen pressure (p), the relative stability of complexes Cu_5–$(O_2)_n$ bearing a number of absorbed molecules (n) can be determined by calculating the Helmholtz free energy of formation:[28–31]

$$\Delta H_f(p,T) = F_{\mathrm{Cu_5-(O_2)}_n}(T) - F_{\mathrm{Cu_5}}(T) - n \cdot \mu_{\mathrm{O_2}}(p,T) \tag{S3}$$

where $F_{Cu_5-(O_2)_n}$ and F_{Cu_5} are the Helmholtz free energies of the $Cu_5-(O_2)_n$ complex and the bare Cu_5 cluster, and μ_{O_2} is the chemical potential of molecular oxygen. For an easier interpretation, considering that we are dealing with an open system for which the number of adsorbed O_2 molecules is arbitrary, we use the definition of the thermodynamical potential ω as stated in Ref. 29:

$$\omega(T,\mu_{O_2},n) = \Delta E_{F,\mathrm{corr}}(T) - T\cdot s_{Cu_5-(O_2)_n}(T) + T\cdot s_{Cu_5}(T) - n\cdot\bar{\mu}_{O_2}(p,T). \qquad \text{(S4)}$$

A (p,T)-phase diagram for Cu_5–$(O_2)_n$ complexes can be constructed on the basis of the potential ω. Assuming a constant number N of Cu_5 clusters but arbitrary amounts of molecular oxygen in the system, $\omega(T,\mu_{O_2},n)$, is defined as the grand potential Ω divided by N, which is a function of the temperature T, the (T,p)-dependent part of the chemical potential of molecular oxygen $\bar{\mu}_{O_2}$, and the number of adsorbed O_2 molecules n (see Eq. S4). In a first approximation, the Cu_5 clusters are treated as fully immobilized on support coupled to a heat bath of temperature T and an infinite reservoir of O_2 gas at pressure p. Under these idealized circumstances, the so defined thermodynamic potential $\omega(T,\mu_{O_2},n)$ will become minimal at thermodynamic equilibrium. A dependence on O_2 pressure enters via $\bar{\mu}_{O_2}(p,T)$.

From this, the number n of adsorbed O_2 molecules which minimizes ω for a specified temperature and a given oxygen pressure can be obtained from Eq. S4 as follows. $\Delta E_{F,\mathrm{corr}}$, the first term on the right-hand side, corresponds to the formation energy of Cu_5–$(O_2)_n$ defined as the difference

$$\Delta E_{F,\mathrm{corr}}(T) = E_{Cu_5-(O_2)_n} - E_{Cu_5} - n\cdot E_{O_2} + E_{\mathrm{corr}}(T) \qquad \text{(S5)}$$

In this equation, $E_{Cu_5-(O_2)_n}$ and E_{Cu_5} denote the DFT energies of the oxygen-covered and pure cluster, respectively, and E_{O_2} is the DFT energy of molecular oxygen. They were obtained with the PBE-D3(BJ) functional using the def2-QZVPP basis set, and including a correction for the basis set superposition error. The last term in Eq. S5, E_{corr}, was introduced to account for the zero-point energy, the thermal vibrational contribution (i.e., as coming from the population of excited vibrational states at a given temperature) as well as to account for thermal rotational, and translational terms. All these corrections were taken from the thermochemistry output of

ORCA.[18] The uncorrected formation energies are plotted in the left-hand panel of Supplementary Figure 17b. The graph shows an approximate linear dependence on the particle number at first, but is also indicating pronounced oxygen interaction effects occurring for higher loads.

The next term on the right-hand side of Eq. S4 introduces a correction with respect to the entropy $s_{Cu_5-(O_2)_n}$ of the cluster. Assuming immobilized copper particles, only vibration and electronic excitation or degeneracy can contribute to the entropy. Vibrational contributions (within the harmonic oscillator approximation) have been also taken from the thermochemistry output of ORCA[18] for temperatures from room temperature (298.15 K) to 773 K. Entropy contributions due to spin multiplicity are automatically included, while contributions due to electronic excitation can be fully neglected at the given temperatures.

The last term on the right side of Eq. S4 is the (T,p)-dependent part of the chemical potential $\bar{\mu}_{O_2}$ of molecular oxygen multiplied by the number n of adsorbed molecules. It introduces the pressure dependence and is of the same magnitude as $\Delta E_{F,\mathrm{corr}}$. We write the (T,p) dependent part of the chemical potential of molecular oxygen as[32]

$$\bar{\mu}_{O_2}(p,T) = \Delta h_{O_2}(p_0,T) - T \cdot s_{O_2}(p_0,T) + R \cdot T \ln\left(\frac{p}{p_0}\right), \tag{S6}$$

where the pressure enters through the ratio p/p_0, with the reference oxygen pressure p_0 set to 1 atm (ca. 1033 mbar). The change of enthalpy is given by $\Delta h_{O_2} = h(p_0,T) - h(p_0,T=0\,K)$. For maximum accuracy we take the values for h_{O_2} and s_{O_2} from the NIST database.[33,34] Note that $\mu_{O_2} = E_{O_2} + \tilde{\mu}_{O_2}$ since the (T,P)-independent contribution to the O_2 chemical potential, E_{O_2}, has been moved to the $\Delta E_{F,\mathrm{corr}}(T)$ term in Eq. S5.

The ω potential is represented in Supplementary Figure 17a for selected sets of (p,T) conditions, including those accessible in the experiment. With all terms of Eq. S4 defined we can now calculate $\omega(p,T,n)$ for the experimentally relevant range of $T = 298$ (room temperature) to 773 K and $p = 1$ atm (1033 mbar) to 10^{-10} mbar and determine the phase of lowest energy for each variable pair (p,T). The resulting phase diagram is given in the right-hand panel of Supplementary Figure 17b. It can be observed that the system is switching between 473 and 673 K. Overall, the phase diagram predicts that complexes with 7 and 4 O_2 molecules as the most stable

species at atmospheric pressure and 0.15 mbar, respectively. As in the phase diagram, it can be clearly observed from Supplementary Figure 17a that the Cu_5–$(O_2)_7$ complex is the most stable configuration at standard conditions of temperature and pressure, while the smaller complex Cu_5–$(O_2)_4$ becomes the most stable upon increasing the temperature and decreasing the pressure.

Copper oxide complexes involving the dissociation of O_2 have also been considered in this work. Depending on the size and temperature, complexes with $5 \leq n \leq 9$ and two dissociated O_2 molecules have a Helmholtz free energy of formation in between 2.2 and 3.5 eV below the non-dissociated counterparts. Although the O_2 dissociated complexes would be the "thermodynamic products", the energy barriers are too high (above 4 eV, see Supplementary Figure 2b) for being over-passed at the temperatures used in the experiment (from RT up to 773 K). The formation of O_2 dissociated products would be then thermodynamically allowed but kinetically forbidden.

References

(1) Hill, J.; Royce, D.; Fadley, C.; Wagner, L.; Grunthaner, F. Properties of oxidized silicon as determined by angular-dependent X-ray photoelectron spectroscopy. *Chem. Phys. Lett.* **1976**, *44*, 225 – 231.

(2) The probability of trapping of O_2 reactant species at the physisorption state has been estimated by considering a Maxwell-Boltzmann distribution of their velocities. In this way, the fraction of O_2 molecules with kinetic energy bellow the energy barrier between physisorption and chemisorption states (ca. 0.1 eV) has been calculated (referred to as P). Finally, the fraction of Cu(I) and Cu(II) oxidation states, as arising from O_2 molecules at the chemisorption minima, has been simply multiplied by P (i.e., considering only the O_2 molecules having overpassed the energy barrier of 0.1 eV).

(3) Matthew, J. Surface analysis by Auger and x-ray photoelectron spectroscopy. D. Briggs and J. T. Grant (eds). IMPublications, Chichester, UK and SurfaceSpectra, Manchester, UK, 2003. 900 pp., ISBN 1-901019-04-7, 900 pp. *Surf. Interface Anal.* **2004**, *36*, 1647–1647.

(4) Zhang, R.; McEwen, J.-S. Local environment sensitivity of the Cu K-Edge XANES fea-

tures in Cu-SSZ-13: Analysis from first-principles. *J. Phys. Chem. Lett.* **2018**, *9*, 3035–3042.

(5) Eren, B.; Heine, C.; Bluhm, H.; Somorjai, G. A.; Salmeron, M. Catalyst chemical state during CO oxidation reaction on Cu(111) studied with ambient-pressure X-ray photoelectron spectroscopy and near edge X-ray adsorption fine structure spectroscopy. *J. Am. Chem. Soc.* **2015**, *137*, 11186–11190.

(6) Grioni, M.; Goedkoop, J. B.; Schoorl, R.; de Groot, F. M. F.; Fuggle, J. C.; Schäfers, F.; Koch, E. E.; Rossi, G.; Esteva, J.-M.; Karnatak, R. C. Studies of copper valence states with Cu L_3-edge x-ray-absorption spectroscopy. *Phys. Rev. B* **1989**, *39*, 1541–1545.

(7) Chou, T.-C.; Chang, C.-C.; Yu, H.-L.; Yu, W.-Y.; Dong, C.-L.; Velasco-Vélez, J.-J.; Chuang, C.-H.; Chen, L.-C.; Lee, J.-F.; Chen, J.-M.; Wu, H.-L. Controlling the Oxidation State of the Cu Electrode and Reaction Intermediates for Electrochemical CO_2 Reduction to Ethylene. *Journal of the American Chemical Society* **2020**, *142*, 2857–2867.

(8) Qayyum, M. F.; Sarangi, R.; Fujisawa, K.; Stack, T. D. P.; Karlin, K. D.; Hodgson, K. O.; Hedman, B.; Solomon, E. I. L-Edge X-ray Absorption Spectroscopy and DFT Calculations on Cu_2O_2 Species: Direct Electrophilic Aromatic Attack by Side-on Peroxo Bridged Dicopper(II) Complexes. *Journal of the American Chemical Society* **2013**, *135*, 17417–17431.

(9) de Lara-Castells, M. P.; Hauser, A. W.; Ramallo-López, J. M.; Buceta, D.; Giovanetti, L. J.; López-Quintela, M. A.; Requejo, F. G. Increasing the optical response of TiO_2 and extending it into the visible region through surface activation with highly stable Cu_5 clusters. *J. Mater. Chem. A* **2019**, *7*, 7489–7500.

(10) Grimme, S.; Ehrlich, S.; Goerigk, L. Effect of the damping function in dispersion corrected density functional theory. *J. Comp. Chem.* **2011**, *32*, 1456–1465.

(11) Grimme, S.; Antony, J.; Ehrlich, S.; Krieg, H. A consistent and accurate ab initio parametrization of Density Functional Dispersion correction (DFT-D) for the 94 elements H-Pu. *J. Chem. Phys.* **2010**, *132*, 154104.

(12) de Lara-Castells, M. P.; Mitrushchenkov, A. O.; Stoll, H. Combining density functional and incremental post-Hartree-Fock approaches for van der Waals dominated adsorbate-surface interactions: Ag_2/graphene. *J. Chem. Phys.* **2015**, *143*, 102804.

(13) de Lara-Castells, M. P.; Bartolomei, M.; Mitrushchenkov, A. O.; Stoll, H. Transferability and accuracy by combining dispersionless density functional and incremental post-Hartree-Fock theories: noble gases adsorption on coronene/graphene/graphite surfaces. *J. Chem. Phys.* **2015**, *143*, 194701.

(14) Bader, R. F. W. A quantum theory of molecular structure and its applications. *Chem. Rev.* **1991**, *91*, 893–928.

(15) Celani, P.; Werner, H.-J. Multireference perturbation theory for large restricted and selected active space reference wave functions. *J. Chem. Phys.* **2000**, *112*, 5546–5557.

(16) de Lara-Castells, M. P.; Cabrillo, C.; Micha, D. A.; Mitrushchenkov, A. O.; Vazhappilly, T. Ab initio design of light absorption through silver atomic cluster decoration of TiO_2. *Phys. Chem. Chem. Phys.* **2018**, *20*, 19110–19119.

(17) López-Caballero, P.; Ramallo-López, J. M.; Giovanetti, L. J.; Buceta, D.; Miret-Artés, S.; López-Quintela, M. A.; Requejo, F. G.; de Lara-Castells, M. P. Exploring the properties of Ag_5–TiO_2 interfaces: stable surface polaron formation, UV-Vis optical response, and CO_2 photoactivation. *J. Mater. Chem. A* **2020**, *8*, 6842–6853.

(18) Neese, F. Software update: the ORCA program system, version 4.0. *Wiley Interdiscip. Rev.: Comput. Mol. Sci.* **2018**, *8*, e1327.

(19) Weigend, F.; Ahlrichs, R. Balanced basis sets of split valence, triple zeta valence and quadruple zeta valence quality for H to Rn: Design and assessment of accuracy. *Phys. Chem. Chem. Phys.* **2005**, *7*, 3297–3305.

(20) Boys, S.; Bernardi, F. The calculation of small molecular interactions by the differences of separate total energies. Some procedures with reduced errors. *Mol. Phys.* **1970**, *19*, 553–566.

(21) Werner, H. J.; Knowles, P. J.; Knizia, G.; Manby, F. R.; Schütz, M.; Celani, P.; Korona, T.; Lindh, R.; Mitrushchenkov, A. O.; Rauhut, G.; et al., MOLPRO, the most recent version, a package of *ab initio* programs, see http://www.molpro.net.

(22) Woon, D. E.; Dunning, Jr., T. H. Gaussian basis sets for use in correlated molecular calculations. Calculation of static electrical response properties. *J. Chem. Phys.* **1994**, *100*, 2975–2988.

(23) Figgen, D.; Rauhut, G.; Dolg, M.; Stoll, H. Energy-consistent pseudopotentials for group 11 and 12 Atoms: Adjustment to multi-configuration Dirac-Hartree-Fock data. *Chem. Phys.* **2005**, *311*, 227–244, Relativistic effects in heavy-element chemistry and physics. In Memoriam Bernd A. Hess (1954–2004).

(24) Zanchet, A.; López-Caballero, P.; Mitrushchenkov, A. O.; Buceta, D.; López-Quintela, M. A.; Hauser, A. W.; de Lara-Castells, M. P. On the stability of Cu_5 catalysts in air using multireference perturbation theory. *J. Phys. Chem. C* **2019**, *123*, 27064–27072.

(25) Mulliken, R. S. Criteria for the construction of good self-consistent-field molecular orbital wave functions, and the significance of LCAO-MO population analysis. *J. Chem. Phys.* **1962**, *36*, 3428–3439.

(26) Hirshfeld, F. L. Bonded-atom fragments for describing molecular charge densities. *Theor. Chim. Acta* **1977**, *44*, 129–138.

(27) Bultinck, P.; Van Alsenoy, C.; Ayers, P. W.; Carbó-Dorca, R. Critical analysis and extension of the Hirshfeld atoms in molecules. *J. Chem. Phys.* **2007**, *126*, 144111.

(28) Yu, X.; Oganov, A. R.; Zhu, Q.; Qi, F.; Qian, G. The stability and unexpected chemistry of oxide clusters. *Phys. Chem. Chem. Phys.* **2018**, *20*, 30437–30444.

(29) Hauser, A. W.; Gomes, J.; Bajdich, M.; Head-Gordon, M.; Bell, A. T. Subnanometer-sized Pt/Sn alloy cluster catalysts for the dehydrogenation of linear alkanes. *Phys. Chem. Chem. Phys.* **2013**, *15*, 20727–20734.

(30) Bhattacharya, S.; Levchenko, S. V.; Ghiringhelli, L. M.; Scheffler, M. Stability and metastability of clusters in a reactive atmosphere: Theoretical evidence for unexpected stoichiometries of $Mg_M\mathbf{O}_x$. *Phys. Rev. Lett.* **2013**, *111*, 135501.

(31) Xu, Y.; Shelton, W. A.; Schneider, W. F. Thermodynamic equilibrium compositions, structures, and reaction energies of Pt_xO_y ($x = 1 - 3$) clusters predicted from first principles. *J. Phys. Chem. B* **2006**, *110*, 16591–16599.

(32) Persson, K. A.; Waldwick, B.; Lazic, P.; Ceder, G. Prediction of solid-aqueous equilibria: Scheme to combine first-principles calculations of solids with experimental aqueous states. *Phys. Rev. B* **2012**, *85*, 235438.

(33) P. J. Linstrom and W. G. Mallard, Eds., NIST Chemistry WebBook, NIST standard reference database number 69, National Institute of Standards and Technology, Gaithersburg MD, 20899, https://doi.org/10.18434/T4D303, (retrieved November 26, 2019).

(34) Chase, M. W., Jr., NIST-JANAF Themochemical Tables, Fourth Edition, J. Phys. Chem. Ref. Data, Monograph 9, 1998, 1-1951.
